# Novel Wide‐Spectrum Virucidal Lipid Nanoparticles

**DOI:** 10.1002/smll.202507669

**Published:** 2025-11-14

**Authors:** Yugo Araújo Martins, Louise Bondeelle, Arnaud Charles‐Antoine Zwygart, Thais Melquiades de Lima, Juliano de Paula Souza, Han Kang Tee, Fernando Chahud, Eurico de Arruda Neto, Francesco Stellacci, Renata Fonseca Vianna Lopez, Caroline Tapparel

**Affiliations:** ^1^ Department of Microbiology and Molecular Medicine Faculty of Medicine University of Geneva Geneva 1211 Switzerland; ^2^ Department of Pharmaceutical Sciences School of Pharmaceutical Sciences of Ribeirão Preto University of São Paulo Ribeirão Preto SP 14040‐900 Brazil; ^3^ Department of Molecular and Cell Biology School of Medicine of Ribeirão Preto University of São Paulo Ribeirão Preto SP 14040‐900 Brazil; ^4^ Department of Pathology School of Medicine of Ribeirão Preto University of São Paulo Ribeirão Preto SP 14040‐900 Brazil; ^5^ Institute of Materials École Polytechnique Fédérale de Lausanne Lausanne 1015 Switzerland

**Keywords:** coronavirus, COVID‐19, nanocarriers, pulmonary delivery, respiratory infection

## Abstract

Viral infections remain a global health challenge, highlighting the urgent need for innovative antiviral strategies. Broad‐spectrum antivirals offer a promising solution. Virustatic compounds fail due to their reversible mechanisms, while existing virucidal agents are frequently limited by toxicity. Here, POSTAN, a novel, biocompatible virucidal lipid nanoparticle engineered for direct antiviral activity is presented. Composed of polyoxyethylene sorbitan oleate (PO) and sodium taurodeoxycholate (ST), POSTAN mimics heparan sulfate (HS) proteoglycans and lipid rafts—host cell structures commonly exploited by viruses for attachment. POSTAN demonstrates optimal physicochemical properties for pulmonary delivery, minimal to no toxicity in Vero cells and human airway epithelial (HAE) cultures, and a favorable safety profile in neonatal mice. It exhibits broad‐spectrum virucidal activity at micromolar concentrations against herpes simplex virus (HSV), respiratory syncytial virus (RSV), Zika virus, Chikungunya virus (CHIKV), and SARS‐CoV‐2 by disrupting viral envelopes. In HAE cultures, POSTAN reduced SARS‐CoV‐2 titers by 5‐ and 3‐log before and after infection. In a neonatal RSV mouse model, intranasal POSTAN led to 6‐, 10‐, and 19‐fold reductions in lung viral titers following prophylactic, therapeutic, or combined prophylactic and therapeutic treatments. It mitigated lung pathology and prevented hemorrhage. These findings support POSTAN as a safe, effective, broad‐spectrum antiviral platform for respiratory infections.

## Graphical abstract

POSTAN is a novel broad‐spectrum antiviral nanoparticle that combines safety with potent virucidal activity. Composed of biocompatible lipids, it mimics heparan sulfate proteoglycans and lipid rafts exploited by viruses, thereby dismantling viral envelopes and blocking replication. Its efficacy in relevant tissue culture models and in vivo highlights its translatability to the clinic.

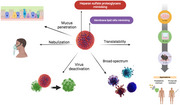

## 1 Introduction

Viruses impact various facets of human life, causing substantial losses in agricultural and livestock production and threatening human health.^[^ ^1^, ^2^, ^3^ ^]^ The management of viral diseases is essential to limit these burdens.^[^ ^4^, ^5^ ^]^ Vaccines are the most effective approach for preventing viral infections, whereas antivirals are necessary after an infection has been established.^[^ ^6^ ^]^ Common challenges encountered with vaccines and antiviral drugs include their specificity for a given virus and their susceptibility to the development of resistance.^[^ ^6^ ^]^ The development of broad‐spectrum compounds that target replication mechanisms shared by many viruses could address these issues.

One strategy for developing such broad‐spectrum antivirals is to mimic widely used viral attachment receptors (VARs), such as heparan sulfate (HS) proteoglycans.^[^ ^7^, ^8^, ^9^ ^]^ HS proteoglycans are negatively charged, branched polysaccharides ubiquitously expressed on the surface of mammalian cells.^[^ ^9^, ^10^ ^]^ Viral–HS interactions increase the concentration of virions at the cell surface, increasing the likelihood that the viruses will encounter a more specific entry receptor.^[^ ^9^, ^11^ ^]^ HS proteoglycans are hijacked by many viruses, including herpes simplex virus (HSV), human papillomavirus, hepatitis C virus, dengue virus, respiratory syncytial virus (RSV), and SARS‐CoV‐2.^[^ ^9^, ^11^, ^12^, ^13^, ^14^, ^15^, ^16^ ^]^ In addition to HS, mimicry of lipid rafts present in the plasma membrane can contribute to the successful development of broad‐spectrum antivirals.^[^ ^17^, ^18^ ^]^ Lipid rafts are nanoscopic, cholesterol‐ and sphingolipid‐rich domains that facilitate viral fusion or endocytosis.^[^ ^17^, ^18^, ^19^, ^20^ ^]^ The involvement of these proteins in these processes is underscored by their consistent association with viral entry receptors and their colocalization with viral glycoproteins and particles.^[^ ^21^, ^22^, ^23^ ^]^

Since 1960, heparin‐like macromolecules and polymers derived from natural or synthetic sources have been used to mimic HS and inhibit a broad spectrum of HS‐dependent viruses.^[^ ^7^, ^8^, ^9^ ^]^ However, such compounds present limitations that hinder their clinical application. Their synthesis or extraction is challenging, and due to their anticoagulant activities, surfactant properties, and short half‐lives, they can cause toxicity at the site of administration, pose pharmacokinetic challenges, and lead to low bioavailability in vivo.^[^ ^7^, ^8^ ^]^ Moreover, their reversible or virustatic mechanism of action further limits their translational value. When these compounds are diluted below a critical concentration, such as in the presence of body fluids, they tend to dissociate from viruses; thus, the viruses can regain infectivity.^[^ ^24^, ^25^, ^26^ ^]^

To address these issues and ensure successful translation for clinical application, it is necessary to develop HS‐mimicking antivirals that achieve irreversible or virucidal activity and remain effective even upon dilution. In parallel, these compounds should be engineered to avoid the toxicity commonly observed with virucidal agents. Modified nanoparticles and nanomaterials functionalized with hydrophobic alkyl chains and VAR‐mimicking moieties have been synthesized, and their antiviral efficacy against a wide range of HS‐dependent viruses has been demonstrated, including HSV, RSV, DENV, ZIKV, and bacteriophages T1, T4, and T7.^[^ ^27^, ^28^, ^29^, ^30^, ^31^, ^32^, ^33^ ^]^ These antivirals generally incorporate rigid multivalent cores, such as silver nanoparticles,^[^ ^34^ ^]^ gold nanoparticles,^[^ ^28^, ^31^, ^32^, ^35^ ^]^ iron nanoparticles,^[^ ^36^ ^]^ silica nanoparticles,^[^ ^27^, ^37^ ^]^ cyclodextrins,^[^ ^29^ ^]^ branched polyglycerols,^[^ ^30^ ^]^ quantum dots,^[^ ^38^, ^39^ ^]^ or graphene sheets,^[^ ^33^ ^]^ and their chemical synthesis typically requires the use of organic solvents.^[^ ^27^, ^28^, ^29^, ^33^ ^]^ These materials are usually unsuitable for the transport and delivery of other biological agents. Moreover, the inhibition of spike‐bearing viruses such as SARS‐CoV‐2 by these sulfated aliphatic‐modified compounds has been shown to be reversible.^[^ ^40^ ^]^

**Figure 1:**
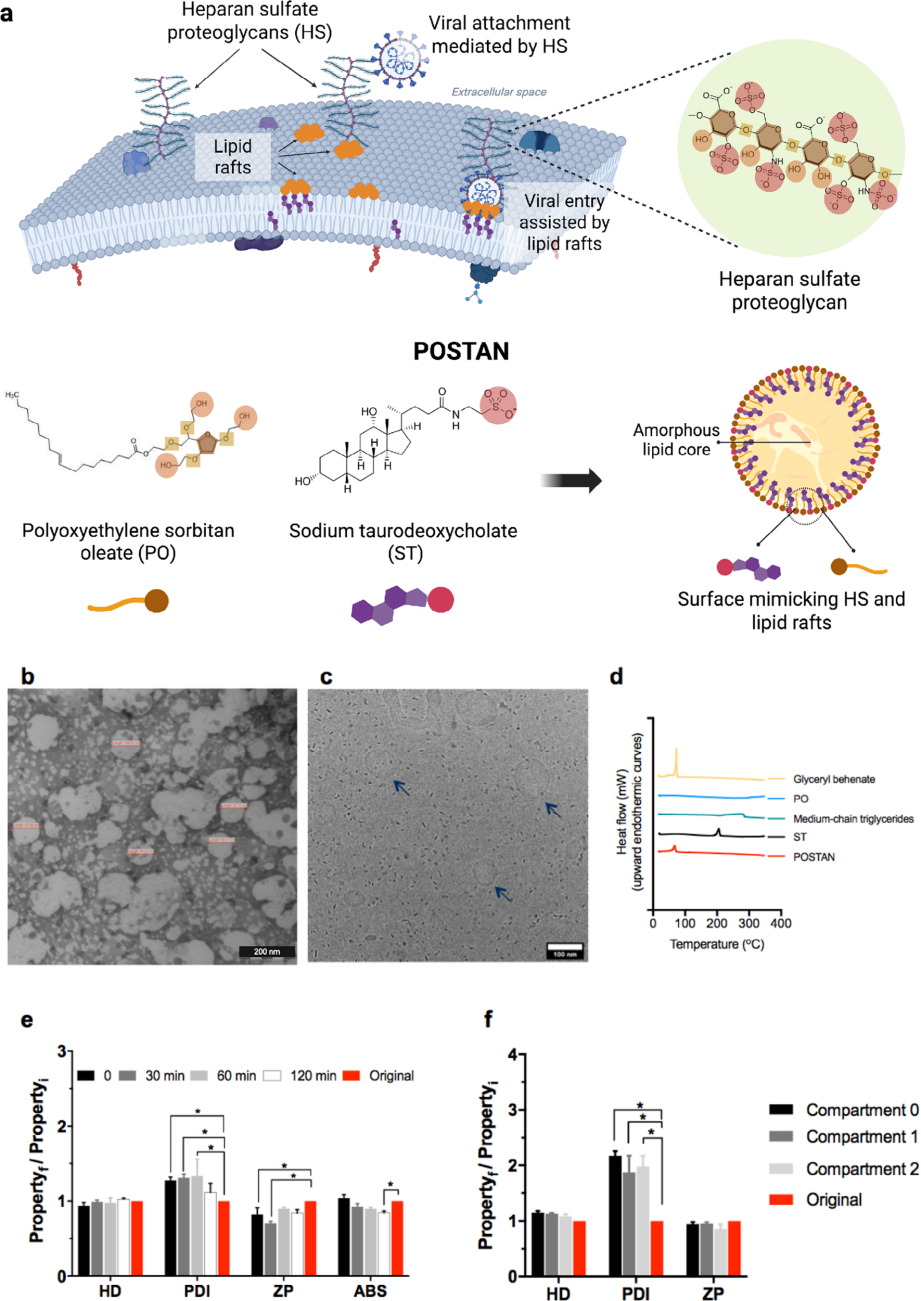
POSTAN characterization: a) Schematic of POSTAN mimicking viral attachment receptors (VARs). The upper panel shows how viruses exploit HS proteoglycans and lipid rafts present at the plasma membrane for cell entry. A magnified view of the HS proteoglycans highlights distinct structural characteristics. The lower panel illustrates POSTAN engineered with polyoxyethylene sorbitan oleate (PO) and sodium taurodeoxycholate (ST) and their key structural moieties assembled on a lipid core, which mimics HS and lipid raft features; b) TEM image (200 nm scale bar); c) Cryo‐EM image (100 nm scale bar); d) DSC thermograms of lyophilized POSTAN and its isolated excipients; e) Evaluation of mucin‒POSTAN interactions via hydrodynamic diameter (HD), polydispersity index (PDI), zeta potential (ZP), and absorbance (ABS) measurements at different times after incubation. HD, PDI, and ZP measurements of POSTAN prior to mucin incubation were used as controls (*n* = 3). The absorbance of the mucin dispersion alone at 650 nm was minimal (0.012 ± 0.001), whereas the initial absorbance of POSTAN was significantly greater (1.2 ± 0.09). f) HD, PDI, and ZP measurements of jet‐nebulized POSTAN in the upper stages (compartments 0 and 1) and lower stage (compartment 2) of the twin‐stage impinger. HD, PDI, and ZP measurements of POSTAN prior to nebulization were used as controls (*n* = 6). The data are expressed as the means ± SDs. Significance was assessed via two‐way ANOVA and Tukey's or Dunnett's test (^*^ *p* < 0.05). The ratio *Property _f_/Property _i_* represents the value of HD, PDI, ZP, or ABS measured after incubation with mucin or after nebulization (*f* = final), divided by the value measured before treatment (*i* = initial).

Here, we report the development of novel biocompatible nanoparticles targeting HS‐dependent viruses that can further serve as versatile antiviral delivery platforms. These newly developed nanoparticles are synthesized via organic solvent‐free approaches and feature several key modifications compared with previous virucidal agents. First, the conventional rigid cores were replaced with an amorphous lipid matrix comprising glyceryl dibehenate and medium‐chain triglycerides, enabling the encapsulation of large concentrations of therapeutic molecules. Second, the ligands used in earlier formulations were replaced with a combination of polyoxyethylene sorbitan oleate (PO), a nonionic emulsifier that stabilizes nanoparticles, and sodium taurodeoxycholate (ST), an endogenous sulfated cholesterol derivative of human bile salts, further contributing to nanoparticle stabilization. The PO and ST combination structurally mimics HS and cholesterol‐rich membrane rafts (**Figure** **1**a), thus enhancing its interactions with viral particles.^[^ ^21^, ^22^, ^23^ ^]^ The resulting antiviral nanoparticles (AN) are referred to as POSTAN. Third, unlike earlier compounds in which sulfated hydrophobic alkyl chains were linked on the nanoparticle surfaces, in POSTAN, the hydrophobic moieties of PO and ST are embedded within the lipid core. As a result, only the hydrophilic VARs remain exposed on the surface (Figure 1a). We hypothesized that, given its sulfated cholesterol‐based composition, POSTAN would better mimic HS in membrane lipid rafts and demonstrate enhanced broad‐spectrum virucidal activity.

In this study, we report the robust and reproducible synthesis of POSTAN through a solvent‐free approach. We show that the compound exhibits broad‐spectrum virucidal activity by disrupting the viral envelope of HS‐dependent viruses, including HSV‐2, ZIKV, CHIKV, and RSV‐A. In a more relevant ex vivo model, we highlight the prophylactic and therapeutic efficacy of POSTAN against SARS‐CoV‐2. Finally, we demonstrate the effectiveness of POSTAN in vivo against human RSV‐A, underscoring its potential for future clinical translation.

## 2 Results and Discussion

### 2.1 Characterization of POSTAN Physicochemical Properties, Nebulization Potential, and Interaction with Mucin

We synthesized POSTAN via melting homogenization coupled with ultrasonication, utilizing glyceryl dibehenate, medium‐chain triglycerides, and PO, which are all FDA‐approved^[^ ^41^ ^]^ and listed in the European STEP database for excipients in pediatric medicines.^[^ ^42^, ^43^ ^]^ The excipients and their concentrations were chosen according to published data and the regulatory agencies’ excipients database^[^ ^41^, ^44^ ^]^ to ensure suitability for topical, pulmonary, and intravenous administration.

Visually, the POSTAN formulation was whitish and displayed macroscopic homogeneity. Dynamic light scattering (DLS) measurements revealed a highly uniform hydrodynamic diameter (HD) (≈ 105 ± 8 nm), a polydispersity index (PDI) of 0.15 ± 0.01, and a negative zeta potential (ZP) of −22 ± 2 mV. Nanoparticle tracking analysis (NTA) revealed that the POSTAN formulation contained 4.3 ± 1 × 10^12^ particles mL^−1^. Representative correlograms from DLS, along with the corresponding plots from ZP and NTA analyses, are provided in the Figure S1 (Supporting Information).

The HD measured by NTA (189 ± 51 nm) was higher than that obtained by DLS (*p* < 0.05) (Table S1, Supporting Information). This discrepancy is well‐documented^[^ ^45^, ^46^, ^47^, ^48^ ^]^ and reflects the distinct principles of each technique: NTA tracks individual particles, allowing for the detection of secondary peaks (Figure S1c, Supporting Information) and generating a number‐weighted size distribution where each particle contributes equally, while DLS provides an intensity‐weighted distribution influenced by scattering intensity, which disproportionately emphasizes a small number of larger particles.^[^ ^45^, ^47^ ^]^ Nevertheless, transmission electron microscopy (TEM) (Figure 1b) and cryogenic electron microscopy (cryo‐EM) (Figure 1c) revealed a spherical shape with a diameter consistent with the DLS data (Table S1, Supporting Information). Of note, the aggregates visible in the TEM images result from the high nanoparticle concentration required for TEM visualization (Figure S1e, Supporting Information). Heat generated by the electron beam can induce partial melting and aggregation of lipid nanoparticles.^[^ ^49^, ^50^ ^]^

Differential scanning calorimetry (DSC) thermograms revealed a single endothermic event corresponding to the melting temperature of glyceryl dibehenate at 73 °C (Figure 1d), suggesting that the melting behavior of the lipid matrix was governed primarily by the properties of this solid lipid.

FITC‐loaded POSTAN (F‐POSTAN) was prepared to evaluate nanoparticle cellular internalization; F‐POSTAN maintained the original physicochemical properties of POSTAN and exhibited an encapsulation efficiency of 93 ± 1.5% (Table S1, Supporting Information). A sustained FITC release profile was observed, with a cumulative release of 9 ± 1% within the first hour, increasing to 34 ± 12% after 24 h in PBS/sodium lauryl sulfate (SLS) receptor medium (Figure S2, Supporting Information). After 24 h, the HD and PDI of the nanoparticles remained stable. The only significant change was in the ZP, which shifted to −32 ± 3 mV due to the presence of SLS in the PBS (*p* < 0.05, Student's *t* test) (Table S2, Supporting Information).

Mucus is the primary respiratory tract barrier to pulmonary‐administered nanosystems. Thus, we analyzed the interactions between POSTAN and mucins (Figure 1e). The HD of POSTAN did not significantly change after incubation with mucin (*p* > 0.05), whereas the PDI increased by 0.03 units after 1 h (*p* < 0.05) and returned to the original value after 2 h (0.11 ± 0.01) (Figure 1e). The zeta potential of the nanoparticles shifted by +5 and +8 mV at 0 and 30 min (*p* < 0.05), respectively, before returning close to the original values (−20 ± 1 mV) (*p* > 0.05) (Figure 1e). The absorbance of POSTAN at 650 nm decreased by only 0.16 units after 2 h of incubation in the mucin solution, dropping from 1.20 ± 0.08 to 1.04 ± 0.02 (*p* < 0.05). Notably, the absorbance of mucin alone was negligible (0.012 ± 0.001).

Absorbance, HD, and ZP measurements were used to estimate particle‒mucin interactions.^[^ ^51^, ^52^, ^53^ ^]^ The initial absorbance values reflected particle mobility, whereas the subsequent reduction following POSTAN incubation with mucin (Figure 1e) suggested partial immobilization, likely due to mucin adsorption on the nanoparticle surface.^[^ ^51^, ^52^, ^53^ ^]^ Despite this, the HD remained stable, and the PDI and ZP values only slightly changed and subsequently returned to their original values in the presence of mucin (Figure 1e), indicating that adsorption was limited and reversible. This behavior is likely driven by electrostatic repulsion between the negatively charged sulfate groups (SO_4_^−^) of mucins^[^ ^54^ ^]^ and the negatively charged POSTAN surface. This repulsion may prevent strong adhesion to the mucus network, allowing POSTAN to diffuse through the mucus layer without being trapped and cleared by mucociliary action. This characteristic is crucial for POSTAN to achieve effective pulmonary delivery and reach its viral target.

Stability during aerosolization is an important consideration in the development of nanoparticles for pulmonary delivery. Smaller nanoparticles are more efficiently embedded into micron‐sized aerosol droplets, enhancing diffusion and facilitating deeper lung penetration.^[^ ^55^, ^56^ ^]^ Nebulizers typically generate aerosol droplets ranging from 1 to 5 µm in diameter,^[^ ^55^ ^]^ enabling effective deposition in both the conducting airways and alveolar regions.^[^ ^57^ ^]^ This delivery method remains valuable for use in both home settings and emergency rooms.^[^ ^55^, ^58^ ^]^ To evaluate the suitability of POSTAN for pulmonary administration, we assessed its physicochemical properties following nebulization (Figure 1f). The nanoparticle suspension was jet‐nebulized and analyzed in each compartment of the TSI apparatus, which included three stages corresponding to the upper (Compartments 0 and 1) and lower (Compartment 2) airways (Figure S3, Supporting Information). After nebulization, the HD increased by 37 nm, and the ZP increased by 5 mV (Figure 1f). However, these changes were not significant compared with those of the original nanoparticles (*p* > 0.05), demonstrating colloidal stability upon nebulization. The PDI of POSTAN increased from 0.10 to 0.16–0.23 (Figure 1f) (*p* < 0.05) but remained below 0.3, indicating high homogeneity even after jet nebulization. This small increase in the POSTAN PDI is likely due to the formation of a minor fraction of aggregates (≤4%) during nebulization, as observed in the DLS analysis.

These data show that nebulized POSTAN retained its HD and ZP within the optimal range for effective transport through the respiratory mucus mesh.^[^ ^59^, ^60^, ^61^ ^]^ This capability is particularly important, as nanoparticles larger than 500 nm or those with a positive zeta potential are more likely to become trapped in mucus, limiting pulmonary distribution.^[^ ^59^ ^]^ Despite a slight increase in PDI, POSTAN exhibited exceptional physicochemical stability compared with other lipid nanoparticles,^[^ ^52^, ^53^, ^62^, ^63^ ^]^ confirming its potential for pulmonary applications.

### 2.2 Cytotoxicity, Tissue Toxicity, and Cellular Internalization

The toxicity of POSTAN was investigated in Vero cells and HAE tissues via resazurin and LDH assays measuring cell metabolic activity and cell lysis, respectively.

**Figure 2:**
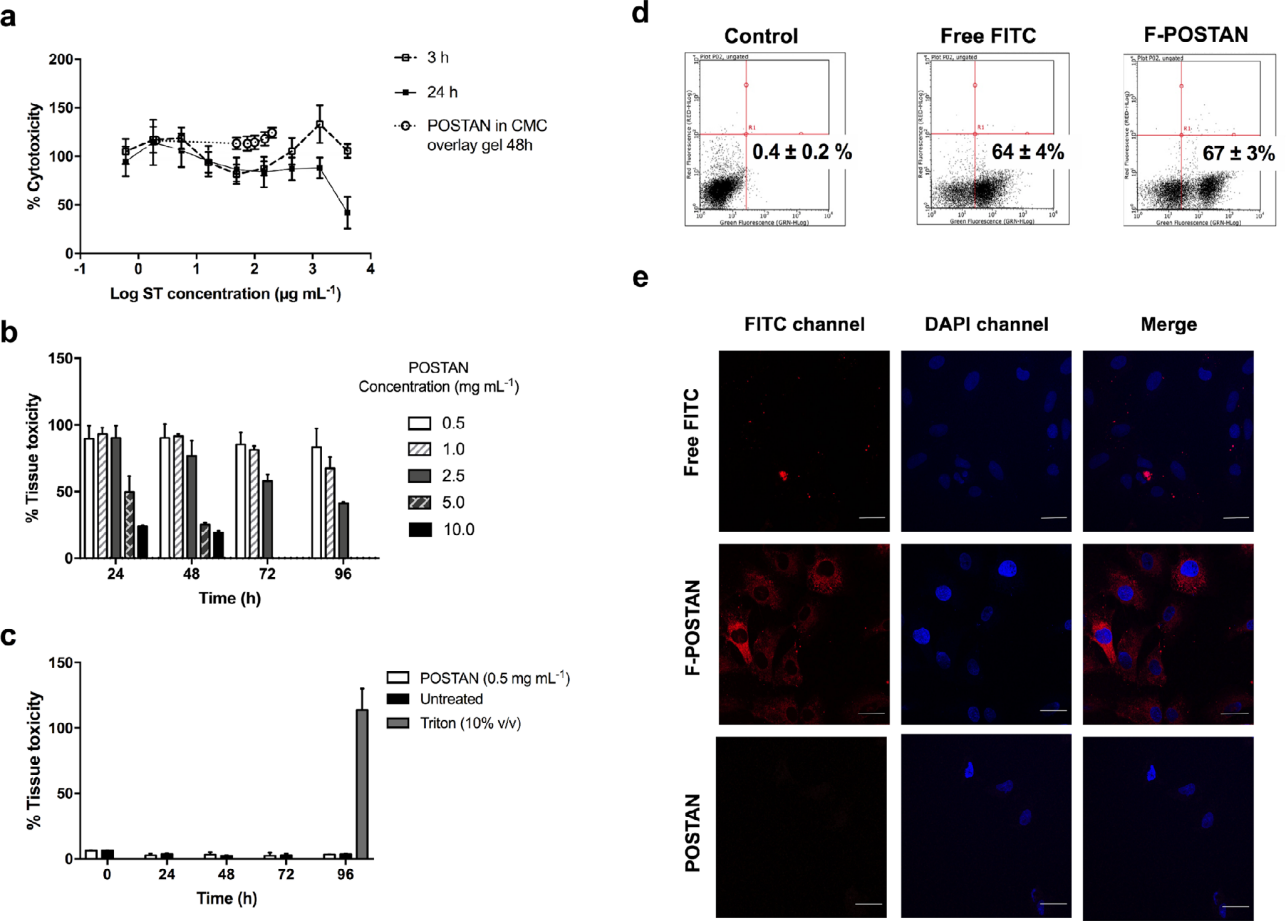
POSTAN cytotoxicity, tissue toxicity, and cellular internalization: a) Vero cell cytotoxicity after exposure to POSTAN for 3 and 24 h and after a 48‐h exposure to POSTAN dispersed in 0.5% CMC overlay gel assessed by resazurin assay (*n* = 3); b) Respiratory tissue metabolic activity after daily exposure to increasing concentrations of POSTAN for 4 days assessed by resazurin assay (*n* = 3); c) Respiratory tissue cell lysis after daily exposure to 0.5 mg mL^−1^ POSTAN for 4 days assessed by LDH assay (*n* = 3); d) Percentage of FITC and F‐POSTAN internalization measured by flow cytometry analysis after incubation with Vero cells for 30 min (*n* = 3). The control group consisted of cells treated with only propidium iodide (PI), a marker used to assess cell integrity. e) Cellular internalization of the FITC solution, F‐POSTAN, and POSTAN after incubation with Vero cells for 30 min, as determined via CLSM. The cell nuclei are stained blue with DAPI, and the FITC fluorescence signal is shown in red (63× magnification with immersion oil; 20 µm scale bar). The data are expressed as the means ± SDs. Significance was assessed via one‐way or two‐way ANOVA and Tukey's test (^*^ *p* < 0.05).

No cytotoxicity was observed in Vero cells at concentrations up to 4000 µg mL^−1^ (3.4 × 10^12^ particles mL^−1^) or 1333 µg mL^−1^ (1.1 × 10^12^ particles mL^−1^) at the two time points used for the efficacy assessment (3 and 24 h of exposure, respectively) (**Figure** **2**a), with a CC_50_ of 1.92 mg mL^−1^ (or 1.65 × 10^12^ particles mL^−1^) after 24 h in Vero cells. Additionally, no cytotoxicity was observed after 48 h of exposure to POSTAN incorporated into the CMC gel at concentrations up to 200 µg mL^−1^ (1.7 × 10^11^ particles mL^−1^) (Figure 2a). The CMC gel retains the nanoparticles above the cells for an extended period, simulating topical administration.

POSTAN up to 1.0 mg mL^−1^ was nontoxic in HAE tissues upon daily administration for 4 days. A 50% reduction in tissue viability was observed at concentrations of ≈5 mg mL^−1^ (or 4.4 × 10^12^ particles mL^−1^) after 1 day and 2.5 mg mL^−1^ (or 2.2 × 10^12^ particles mL^−1^) after 4 days of exposure to the nanoparticles (Figure 2b). The LDH assay further confirmed that daily administration of 0.5 mg mL^−1^ POSTAN (or 4.3 × 10^11^ particles mL^−1^) had no toxic effects over 4 days (Figure 2c).

We next checked the ability of POSTAN to cross cell membranes. Free FITC was used as a positive control. The internalization levels of the cells incubated with FITC and F‐POSTAN were comparable (62 ± 4% and 52 ± 6%, respectively) (Figure 2d). Confocal laser scanning microscopy (CLSM) analysis (Figure 2e) revealed that the red fluorescence in F‐POSTAN‐treated cells indicated efficient nanoparticle internalization, with widespread distribution throughout the cytoplasm. The cells treated with free FITC were internalized; however, the intracellular distribution was limited. As expected, cells treated with nonlabeled POSTAN showed no fluorescence and maintained a normal morphology, further confirming the safety of the nanoparticles. The efficient internalization of POSTAN into cells highlights its potential use as a drug delivery platform for intracellularly active agents, such as conventional antivirals or anti‐inflammatory drugs.

Notably, F‐POSTAN presented an encapsulation efficiency of 93% (Table S1, Supporting Information) and retained most of the FITC marker in the cellular internalization assay. Only 10% of the FITC was released into the medium after 1 h (Figure S2, Supporting Information), likely corresponding to the small fraction of nonencapsulated FITC present in the formulation. Since the cellular internalization assay was conducted with F‐POSTAN and FITC at the same concentration (1 mg mL^−1^) and the exposure time was limited to 30 min, the fluorescence signal observed in the cellular uptake assays can be attributed primarily to the fluorescent nanoparticles.

The saturation of F‐POSTAN uptake after 30 min of incubation (unpublished data), together with the nanoparticle's negative surface ZP, reduces their affinity for the cell membrane and leaves them available extracellularly to interact with the viral particles. These factors suggest that a substantial fraction of nanoparticles remains extracellular and available to interact with viral particles, thereby contributing to the observed antiviral activity.

### 2.3 Antiviral Mechanism of Action

Next, we assessed the antiviral activity of POSTAN. Sodium 10‐undecene‐1‐sulfonate‐grafted cyclodextrins (CD1), an HS mimic with established virucidal activity against HSV‐2,^[^ ^29^ ^]^ was used as a control.

**Figure 3:**
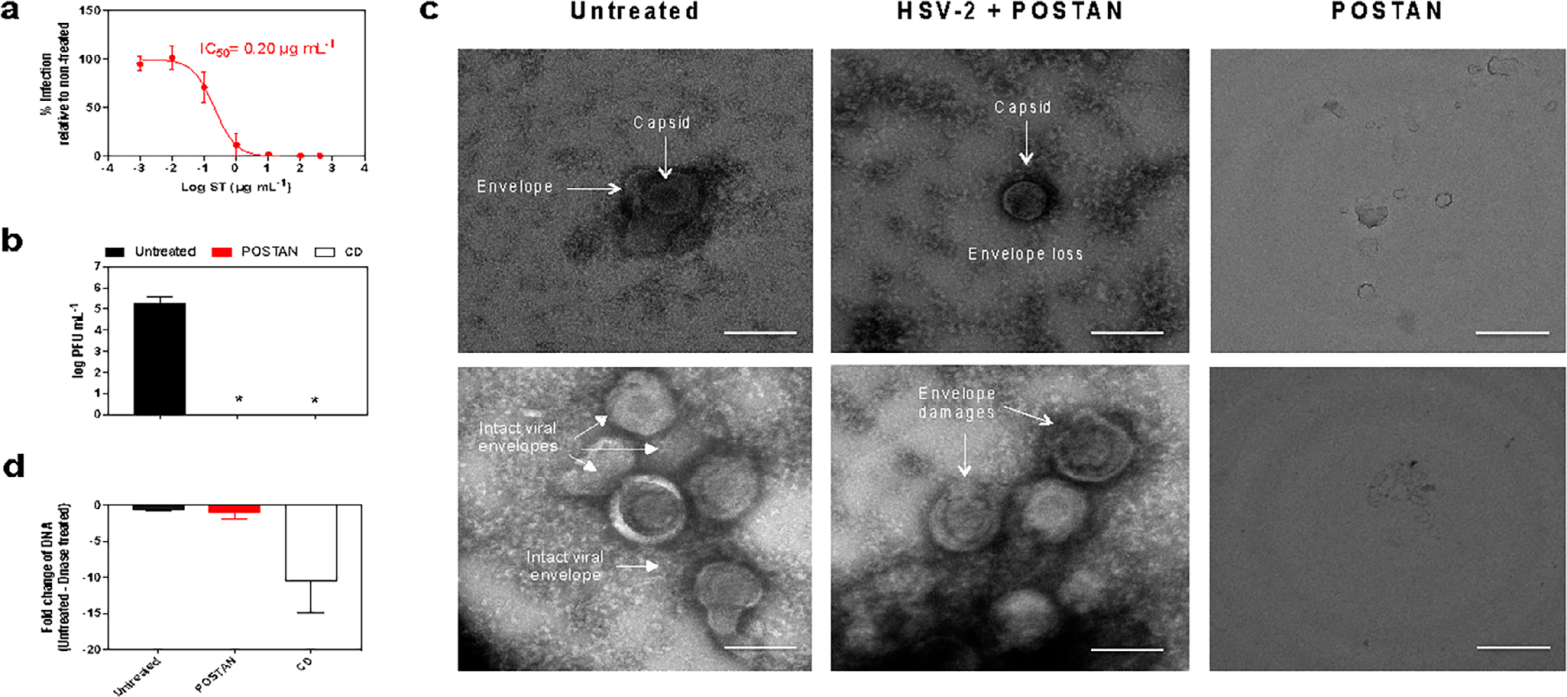
Mechanism of action of POSTAN: a) Dose‒response assay in Vero cells: serial dilutions of POSTAN were incubated with HSV‐2 (MOI of 0.02) for 2 h at 37 °C before addition to cells (virus pretreatment). Viral infectivity inhibition and IC_50_ values were established at 48 hpi via a plaque assay, which compared HSV‐2 treated with nanoparticles to the untreated control (*n* = 4). b) Virucidal assays: HSV‐2 (MOI of 0.1) was incubated with POSTAN (100 µg mL^−1^) or CD1 (100 µg mL^−1^) for 2 h at 37 °C, followed by serial dilution of Vero cells. Viral titers of untreated and treated HSV‐2 were measured via plaque assays (*n* = 3). c) TEM images of HSV‐2 (4.5 × 10^9^ PFU mL^−1^) and HSV‐2 treated with POSTAN (10 µg mL^−1^) for 2 h at 37 °C; 50× magnification and 100 nm scale bars. d) DNA exposure assay: HSV‐2 (10^5^ PFU mL^−1^) was treated with POSTAN (100 µg mL^−1^) or CD1 at a concentration 500 times greater than the IC_50_ for 2 h at 37 °C and then incubated for 30 min with Turbo DNase or buffer only. DNA was then extracted and subjected to qPCR (*n* = 3). The data are expressed as the means ± SDs. Significance was assessed via one‐way ANOVA and Dunn's test (^*^ *p* < 0.05).

A dose‒response assay was conducted by preincubating the HSV‐2 virus with the nanoparticles for 2 h prior to infection. POSTAN exhibited an IC_50_ of 0.20 µg mL^−1^ (**Figure** **3**a), which is 132‐fold lower than that of CD1 (26.4 µg mL^−1^)^[^ ^29^ ^]^ and 4‐fold lower than that of acyclovir (0.825 µg mL^−1^)^[^ ^29^ ^]^ against the same virus.

To determine whether the inhibition caused by POSTAN was irreversible, we conducted a virucidal assay as previously described.^[^ ^28^, ^29^ ^]^ HSV‐2 (MOI of 0.1) was first incubated with POSTAN at 100 µg mL^−1^ for 2 h. After the virus/nanoparticle complex was serially diluted to concentrations below the nanoparticle IC_50_, the number of infectious virions was determined via plaque assay. Compounds that induce a clear reduction in virion infectivity, even at concentrations below the IC_50_ values, are considered virucidal, whereas compounds that lose antiviral activity upon dilution are considered virustatic and likely act through competitive binding with the receptor. POSTAN permanently inactivated virions (*p* < 0.05), as evidenced by the absence of infectious viruses (Figure 3b; Figure S4a, Supporting Information). This virucidal activity was comparable to that of CD1.^[^ ^29^ ^]^

To confirm POSTAN's unique virucidal mechanism, two related control nanoparticles (CN1 and CN2) were synthesized. Sodium taurodeoxycholate was replaced with sodium 10‐undecene‐1‐sulfonate (a sulfonated, non‐cholesterol compound) in CN1 and with a tocopherol derivative (a non‐sulfonated, non‐cholesterol compound) in CN2. CN1 exhibited a mean diameter of 102 ± 17 nm, PDI of 0.11 ± 0.01, and zeta potential of –16 ± 2 mV; CN2 displayed a mean diameter of 97 ± 22 nm, PDI of 0.16 ± 0.02, and zeta potential of −11 ± 1 mV. DLS analysis revealed colloidal properties comparable to POSTAN, except for the zeta potential (ZP), which was significantly higher for CN1 and CN2 (*p* < 0.05) (Table S1, Supporting Information).

Dose–response assays revealed that CN1 exhibited IC_50_ value of 0.14 µg mL^−1^ (Figure S4b, Supporting Information), comparable to that of POSTAN (0.2 µg mL^−1^), likely linked to the sulfonate‐mediated electrostatic interactions. However, CN1 was only virustatic (Figure S4b, Supporting Information). This can be attributed to its non‐cholesterol composition rather than its higher zeta potential once it showed viral inhibition. In contrast, CN2 displayed neither antiviral nor virustatic activity, consistent with its inert pegylated surface (Figure S4c, Supporting Information). Collectively, these results confirm that POSTAN's dual‐mimicking strategy, combining heparan sulfate and cholesterol lipid‐raft features, is essential to confer virucidal activity.

To demonstrate the virucidal mechanism of POSTAN via another method, purified HSV‐2 was incubated with POSTAN for 2 h, fixed, and observed via TEM (Figure 3c). In the untreated samples, HSV‐2 retained its typical morphology, with a diameter of ≈100 nm, a capsid, and an envelope. In contrast, virions incubated with POSTAN presented morphological alterations, such as envelope rupture or complete envelope loss (Figure 3c). We further confirmed the virucidal mechanism of action via a DNA exposure assay, as previously conducted with CD1.^[^ ^29^ ^]^ Surprisingly, unlike that observed with CD1, the damage induced by POSTAN was restricted to the viral envelope, and the HSV‐2 genome remained inaccessible to DNase even after exposure to the nanoparticles (Figure 3d). This finding suggests that, in contrast to CD1,^[^ ^29^ ^]^ POSTAN preserves capsid integrity. Nevertheless, disruption of the viral envelope may be sufficient to irreversibly affect viral infectivity by preventing any further interaction between the virus surface protein and the cellular receptor.^[^ ^28^ ^]^

We propose that the action of POSTAN on the viral envelope results from multivalent binding between viral surface proteins and POSTAN HS mimics.^[^ ^37^, ^64^ ^]^ This interaction induces stretching in the viral membrane beyond a critical point, leading to irreversible membrane rupture. The cholesterol derivative (ST) component of the POSTAN platform may play a crucial role in this mechanism, providing both electrostatic and hydrophobic interactions essential for engaging viral particles. Other studies have highlighted the importance of both hydrophobic and electrostatic interactions for effective virus inhibition.^[^ ^29^, ^33^ ^]^

**Figure 4:**
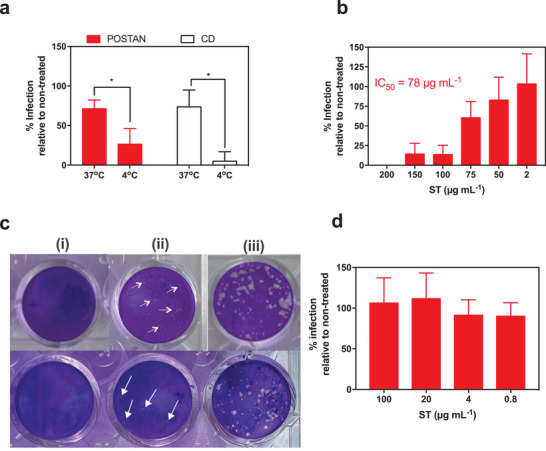
Mechanistic insights into the antiviral action of POSTAN. a) Virus penetration assay: HSV‐2 (MOI of 0.002) and host cells prechilled on ice were incubated at 4 °C for 45 min to allow viral attachment and prevent entry. As a control, a parallel assay was performed at 37 °C. After incubation, the unbound viruses were removed, and POSTAN or CD1 at 100 µg mL^−1^ was added to the cells for 2 h at 37 °C. Viral infectivity inhibition at 48 hpi was quantified via a plaque assay, which compared the cells treated with the compounds to untreated controls for both the 4 and 37 °C conditions (*n* = 4). b) POSTAN therapeutic activity: Increasing concentrations of POSTAN dispersed in the CMC/DMEM overlay were added to the cells for 1 h after HSV‐2 infection (MOI of 0.002) (postinfection treatment). Viral infectivity inhibition and IC_50_ values were established at 48 hpi via a plaque assay, which was used to compare the POSTAN‐treated cells to the untreated controls (*n* = 5). c) Images of HSV‐2 plaques in infected cells treated with 200 µg mL^−1^ (i) and 50 µg mL^−1^ (ii) POSTAN, in contrast to the results in untreated cells (iii). d) Cell‐mediated activity: Vero cells were pretreated with increasing concentrations of POSTAN for 3 h at 37 °C, the nanoparticles were washed out, and the cells were infected with HSV‐2 (MOI of 0.002) (cell pretreatment). Viral infectivity was established at 48 hpi via a plaque assay comparing POSTAN‐treated to untreated control cells (*n* = 3). The data are expressed as the means ± SDs. Significance was assessed via Student's *t* test or one‐way ANOVA with Tukey's test (^*^ *p* < 0.05).

We next investigated whether POSTAN could inhibit viral penetration even when the virion was already attached to host cells (**Figure** **4**a). We incubated the cells and viruses on ice for 1 h to allow virus attachment to the host cell but prevent internalization. After the removal of unbound virions, POSTAN was added, and the temperature was either maintained at 4 °C or shifted to 37 °C to allow virus entry. When POSTAN and CD1 were added during the penetration step (at 37 °C), both inhibited viral entry by ≈25% (Figure 4a). However, the inhibition reached 75% when the nanoparticles were added during attachment (at 4 °C) and before internalization (*p* < 0.05, Student's *t* test) (Figure 4a). These findings confirmed that POSTAN and CD1 act primarily on extracellular viral particles. The ability of POSTAN to outcompete viral binding might be because of its higher affinity for the viral ligand, causing the detachment of virions from the cell membrane and/or altering the viral envelope of bound viruses, which inhibits subsequent entry.^[^ ^37^ ^]^

To obtain a comprehensive overview of the antiviral mechanism of action of POSTAN, we further evaluated its therapeutic potential when added after infection and its possible cell‐mediated effects. In the postinfection treatment study, POSTAN dispersed in a 0.5% CMC gel was added to infected cells for 48 h. The CMC gel retains the nanoparticles above the cells for a prolonged period, simulating topical administration. The plaque reduction assay revealed dose‐dependent inhibition efficacy, with an IC_50_ of 74 µg mL^−1^ (Figure 4b). These findings confirm the therapeutic potential of POSTAN. Interestingly, we observed a reduction in plaque size in the treatment versus control groups (only virus and CMC gel) (Figure 4c), indicating that POSTAN also restrained virion spread to neighboring cells. No inhibitory activity was observed when the cells were preincubated with only POSTAN (Figure 4d), ruling out an indirect cell‐mediated effect and supporting previous data on the extracellular action of the nanoparticles.

To establish whether POSTAN exhibits broad‐spectrum antiviral activity, we investigated its inhibitory effect on several HS‐dependent viruses. As shown in **Table** **1**, POSTAN exhibited IC_50_ values of 48, 101, and 118 µg mL^−1^ against pseudotyped VSV‐∆‐CoV‐2, SARS‐CoV‐2, and RSV‐A, respectively. For comparison, hydroxychloroquine displayed IC_50_ values of 1.31 and 0.98 µg mL^−1^ against VSV‐∆‐CoV‐2 and SARS‐CoV‐2,^[^ ^40^ ^]^ while ribavirin demonstrated an IC_50_ of 10 µg mL^−1^ against RSV‐A (Figure S4d, Supporting Information).

**Table 1:** Inhibitory activity of POSTAN against different viruses.

Virus	CC_50_ Vero (mg mL^−1^)^$^	Number of POSTAN mL^−1^	IC_50_ [µg mL^−1^] (95% CI)	Virucidal activity	Selectivity index
HSV‐2	> 1.9	1.72 x 10^8^	0.2 (0.15–0.29)	+	> 9500
ZIKV	> 1.9	4.98 x 10^9^	6 (1.5–29.1)	+	> 328
CHIKV	> 1.9	1.39 x 10^11^	162 (72.5–346)	+	> 12
RSV‐A	> 1.9	1.01 x 10^11^	118 (29.7–185)	+	> 16
SARS‐CoV‐2 Delta variant	> 1.9	2.14 x 10^11^	249 (168.4–452.2)	+	> 8
SARS‐CoV‐2 Omicron variant	> 1.9	2.35 x 10^11^	101 (73.6–136.9)	+	> 19
Pseudotype VSV‐∆‐CoV‐2	> 1.9	4.13 x 10^10^	48 (18.87–107.7)	n/a	> 40

^$)^ Values calculated after 24 h of incubation with Vero cells; n/a: not assessed.

Although POSTAN shows higher IC_50_ values compared to CD1, its favorable selectivity index (SI) underscores its potential as a promising candidate for clinical development. Whereas CD1 exhibited a CC_50_ above 300 µg mL^−1^,^[^ ^29^ ^]^ POSTAN demonstrated a markedly higher biocompatibility, with an experimentally determined CC_50_ of 1.9 mg mL^−1^. POSTAN demonstrated a selectivity index (SI) greater than 9500 against HSV‐2. For ZIKV, the SI exceeded 328, whereas for CHIKV, RSV‐A, and the SARS‐CoV‐2 Omicron variant, the SI values ranged from > 12 to > 19 (Table 1).

**Figure 5:**
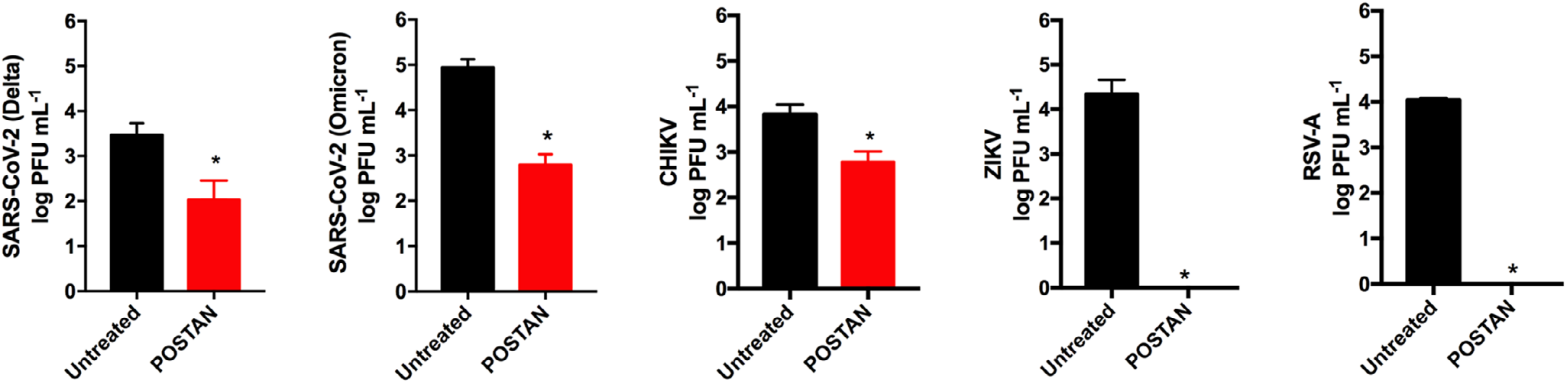
Broad‐spectrum virucidal activity of POSTAN against HS‐dependent viruses. Virucidal assays: SARS‐CoV‐2 Delta (MOI of 1) and Omicron (MOI of 10) variants, CHIKV (MOI of 0.01), ZIKV (MOI of 0.01), and RSV‐A (MOI of 0.1) were incubated with POSTAN at 100 µg mL^−1^ for 2 h at 37 °C, followed by serial dilution in Vero cells. Titers of untreated and POSTAN‐treated viruses were measured via plaque assays (*n* = 3). The data are expressed as the means ± SDs. Significance was assessed via Student's *t* test or the Mann‒Whitney test (^*^ *p* < 0.05).

POSTAN exhibited antiviral activity against a wide range of viruses from different families, including virucidal action against the SARS‐CoV‐2 Delta and Omicron variants from the *Coronaviridae* family, CHIKV from the *Togoviridae* family, ZIKV from the *Flaviviridae* family, and RSV‐A from the *Pneumoviridae* family (**Figure** **5**). Additionally, the nanoparticles demonstrated efficacy against the VSV‐∆‐CoV‐2 pseudotype, which expresses the SARS‐CoV‐2 Omicron variant spike protein (Table 1). These results highlight POSTAN's potent antiviral activity across multiple viruses and demonstrate its strong safety profile.

### 2.4 Ex Vivo Efficacy

Given the observed broad‐spectrum virucidal activity and nontoxicity profile of POSTAN, we tested whether its antiviral activity was preserved in HAE tissue cultures against the SARS‐CoV‐2 Omicron variant. Cultures were derived from human bronchial biopsies and redifferentiated in vitro at the air‒liquid interface to replicate the 3D structure of human respiratory tissues. Once differentiated, the tissues closely mimic the architecture of the upper respiratory airway epithelium, featuring a characteristic mucus layer and mucus‐secreting, ciliated, and basal cells. The presence of mucus and mucociliary clearance, primordial defense mechanisms of our airways, may affect the efficacy of antivirals administered from the apical tissue side.

**Figure 6:**
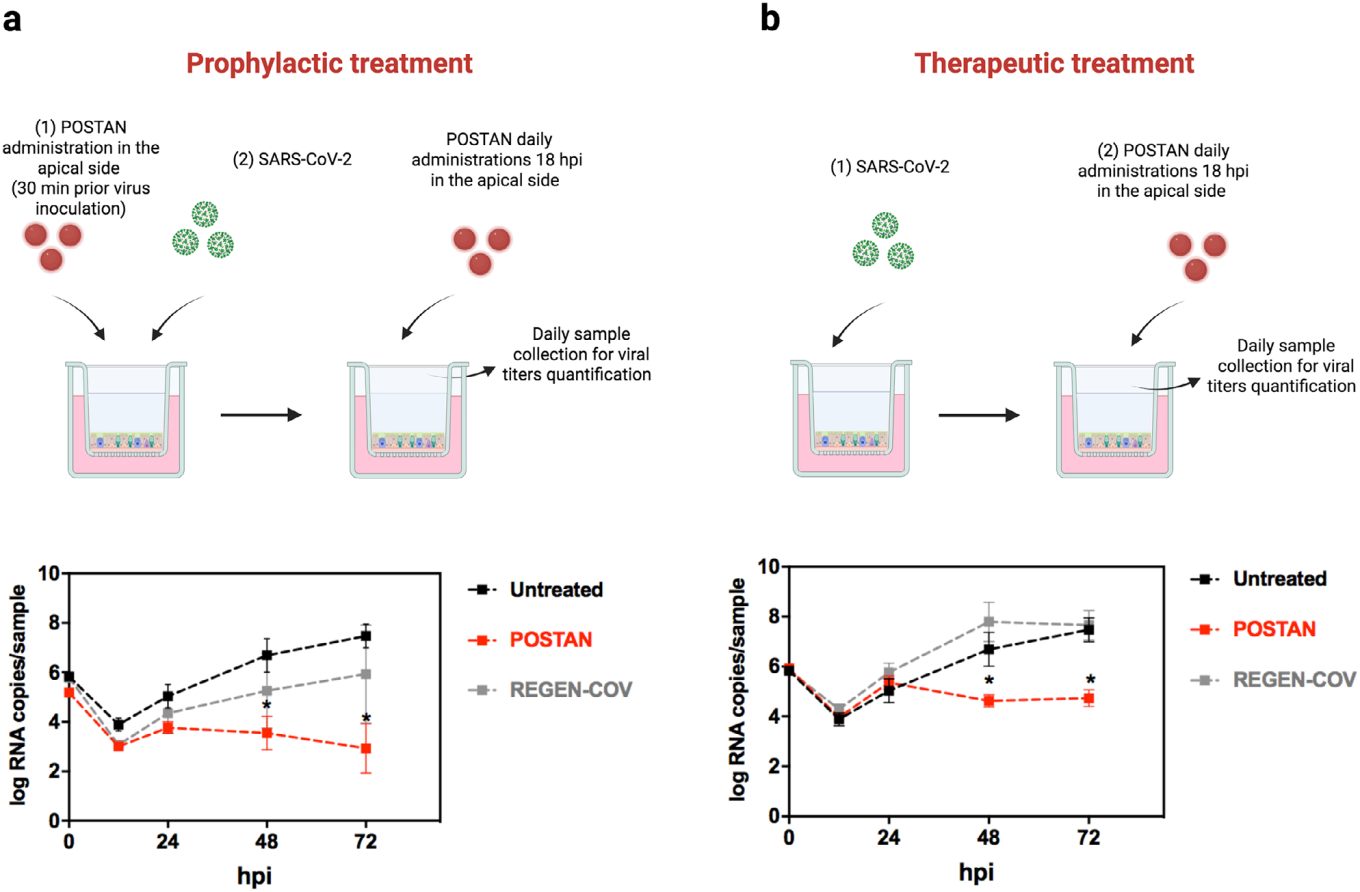
Ex vivo efficacy of POSTAN: a) Prophylactic treatment of respiratory tissues with SARS‐CoV‐2 (4 × 10^4^ PFU mL^−1^) and daily administration of POSTAN (30 µL; 0.5 mg mL^−1^ or 4.3 × 10^11^ particles mL^−1^). Treatment was initiated 30 min prior to virus inoculation (*n* = 4); b) Therapeutic treatment of respiratory tissues with SARS‐CoV‐2 (4 × 10^4^ PFU mL^−1^) and daily administration of POSTAN (30 µL; 0.5 mg mL^−1^ or 4.3 × 10^11^ particles mL^−1^). Treatment was initiated at 18 hpi (*n* = 4). The viral loads of untreated, POSTAN‐treated, and REGEN‐COV‐treated tissues were measured via RT‒qPCR; the data are expressed as the means ± SDs. Statistical significance was assessed via Student's *t* test (^*^ *p* < 0.05).

We tested the prophylactic potential of POSTAN by adding the nanoparticles to the apical site of the tissues 30 min before infection, followed by daily administration, starting at 18 hpi (**Figure** **6**a). 4.0‐ and 4.6‐log reductions in viral titers were observed at 48 and 72 h, respectively (*p* < 0.05, Student's *t* test). We next assessed the therapeutic potential of POSTAN by administering the nanoparticles 18 hpi (Figure 6b). A 2.9‐log reduction in viral titers was observed at 48 and 72 h (*p* < 0.05). As viral replication is halted in both scenarios, these results underscore the preventive and therapeutic efficacy of the nanoparticles and indicate that the antiviral activity observed in vitro is effectively translated to ex vivo human models.

Notably, POSTAN demonstrated superior efficacy to REGEN‐COV, a combination of casirivimab and imdevimab monoclonal antibodies (Figure 6). No significant difference in viral titers was observed between REGEN‐COV‐treated tissues and untreated control tissues in either preventive (Figure 6a) or therapeutic (Figure 6b) assays (*p* > 0.05). REGEN‐COV virus‐neutralizing antibodies bind noncompetitively to the critical receptor‐binding domain of the virus's spike protein, inhibiting its interaction with the human angiotensin‐converting enzyme 2 (ACE2) viral receptor and subsequent infection.^[^ ^65^ ^]^ However, REGEN‐COV has lost efficacy against Omicron variants because of the emergence of mutations in the virus spike protein,^[^ ^66^ ^]^ leading to its deauthorization by the FDA.^[^ ^67^ ^]^ In contrast to monoclonal antibodies, our HS‐mimicking approach targets conserved regions of the viral envelope, offering a broad‐spectrum virucidal effect (Figure 4) that overcomes challenges posed by surface protein mutations, as demonstrated by the conserved susceptibility of the SARS‐CoV‐2 Delta and Omicron variants to POSTAN (Table 1), while also reducing the risk of drug resistance.^[^ ^29^ ^]^

### 2.5 In Vivo Efficacy

POSTAN demonstrated stability during nebulization, low affinity for mucus, broad‐spectrum virucidal activity at micromolar concentrations, and strong efficacy in 3D HAE tissue cultures. These results prompted us to evaluate the antiviral efficacy of the nanoparticles against RSV‐A in a neonatal mouse model.^[^ ^68^ ^]^

When administered intranasally alone, POSTAN exhibited good biocompatibility in healthy neonatal mice. Daily POSTAN intranasal administration for five days did not induce any pathological changes or inflammation in the lungs of the animals, underscoring its safety profile (Figure S5, Supporting Information). In selecting the excipients and their concentrations for POSTAN, we consulted relevant literature and the pharmaceutical excipients database from the Food and Drug Administration^[^ ^41^, ^42^, ^43^, ^44^ ^]^ to ensure the suitability of the formulation for pulmonary administration. The low toxicity of POSTAN, even after daily administration, offers a substantial advantage for its potential use as an antiviral agent, ensuring that the nanoparticles can be applied without causing harm to respiratory epithelial tissues. This property is crucial for developing safe and effective treatments for respiratory viruses.^[^ ^69^, ^70^ ^]^

For the efficacy tests, C57BL/6 neonatal mice were randomly divided into five groups. The experimental design is shown in the (Figure S6, Supporting Information). One group served as the healthy control (i.e., uninfected and untreated), whereas the remaining four groups were intranasally infected with RSV‐A and received the following treatments: a) the untreated group received PBS once daily, starting at 24 hpi; b) the prophylaxis group (P) received a single dose of POSTAN administered 15–25 min before infection, followed by daily PBS for 4 consecutive days; c) the prophylaxis plus treatment group (P+T) received the same prophylactic POSTAN dose prior to infection, followed by daily POSTAN administration for 4 days, continuing treatment at 24 hpi; d) the therapeutic treatment group (TT) received POSTAN once daily for 4 days, starting at 24 hpi. POSTAN was administered intranasally at a concentration of 5 mg mL^−1^ (4.3 × 10^12^ particles mL^−1^). After treatment completion, the mice were euthanized, and their lungs were collected for homogenization and subsequent quantification of RSV‐A.

Neonatal mice exhibit increased susceptibility to RSV infection due to their immature immune system.^[^ ^71^, ^72^, ^73^ ^]^ This model is also considered a relevant surrogate for the human population most at risk, infants under one year of age.^[^ ^74^, ^75^ ^]^ As commonly reported, the neonatal mouse model presents inherent variability in RSV RNA levels among infected controls.^[^ ^68^ ^]^ Despite this variability, treatment‐induced differences remained statistically significant.

**Figure 7:**
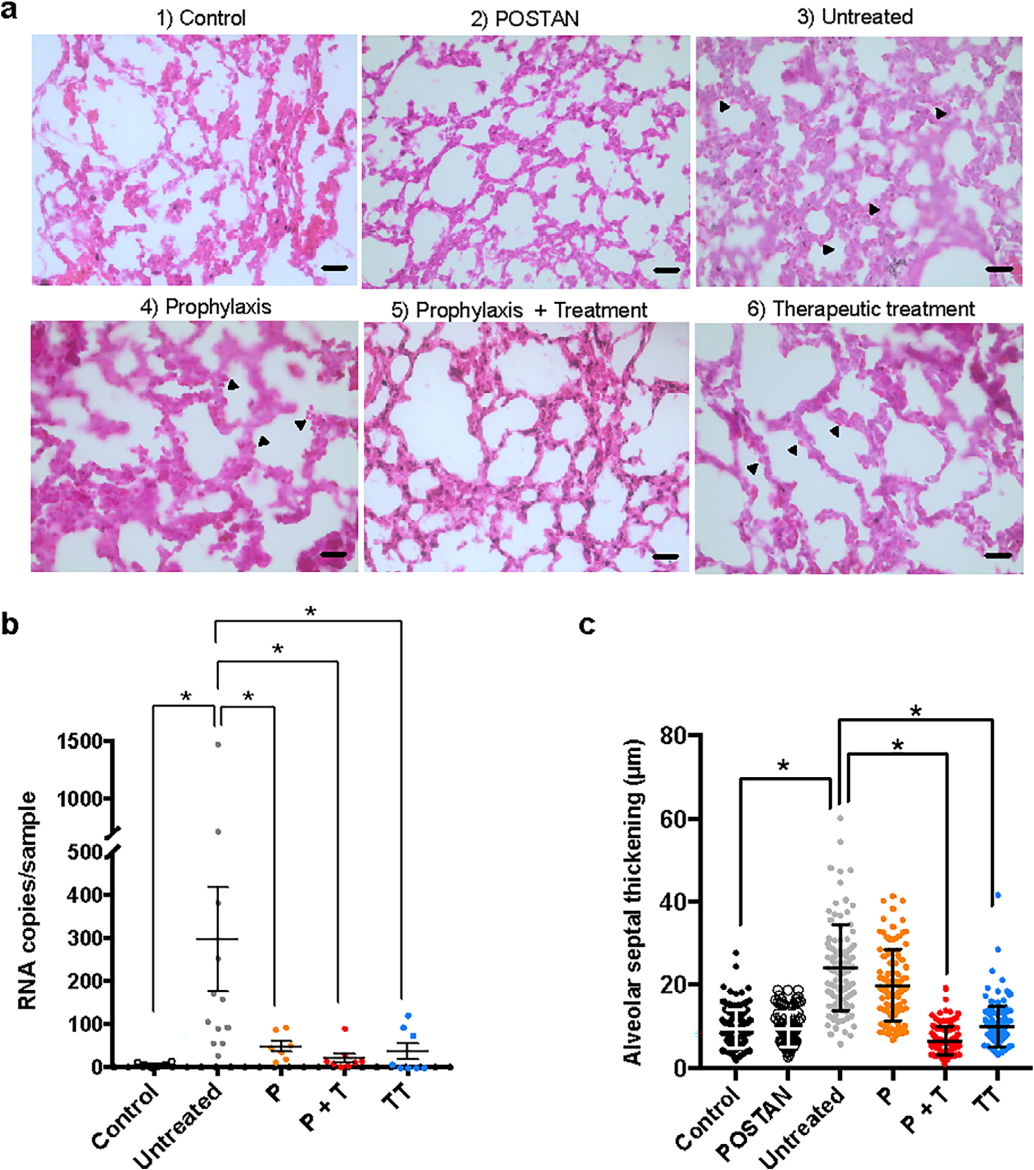
Intranasal RSV‐A infection and POSTAN efficacy in neonatal mice: a) Histological sections of lungs stained with hematoxylin and eosin (20 µm scale bar); the arrows indicate red blood cells; b) Viral titers in the lung tissues of C57BL/6 neonatal mice (*n* = 8–12); c) Measurements of alveolar septum thickening (*n* = 100). The data are expressed as the means ± SDs. The error bars represent the SDs. Significance was assessed via the Kruskal‒Wallis test and Dunn's test or the Mann‒Whitney test (^*^ *p* < 0.01). The mice were intranasally inoculated with RSV‐A (10 µL; 1.7 × 10^5^ PFU mL^−1^, 850 PFU per gram of animal), with 5 µL distributed per nostril. PBS or POSTAN (5 mg mL^−1^ or 4.3 × 10^12^ particles mL^−1^) was administered similarly (5 µL per nostril). Groups: Control ‐ healthy animals (no treatment); POSTAN ‐ 5‐day administration of POSTAN in healthy animals; Untreated ‐ RSV‐A‐infected animals treated with PBS (once per day) for 4 days, starting 24 hpi; P ‐ Single dose of POSTAN administered 15–25 min before infection, followed by 4 days of PBS administration (once per day), starting 24 hpi; P+T ‐ One dose of POSTAN administered 15–25 min before infection, followed by 4 days of POSTAN administration (once per day); TT ‐ POSTAN treatment administered for 4 days, starting 24 hpi.

Compared with noninfected neonatal mice, C57BL/6 neonatal mice infected with RSV‐A did not exhibit weight loss, as observed in the Figure S7, Supporting Information. However, successful RSV inoculation was confirmed at up to 5 days post‐inoculation on the basis of the lung viral load (**Figure** **7**b), which aligns with findings from other studies that detected infectious RSV particles at 7 days post‐inoculation.^[^ ^68^ ^]^ The survival rate of the POSTAN‐treated animals was 100%, whereas one animal in the untreated RSV‐A‐infected group died on day 2.

Compared with those in the untreated group, the viral loads in the lungs of treated mice were significantly lower (Figure 7b). The viral titers in the lungs of the mice pretreated with a single dose of POSTAN (P) were 6 times lower than those in the lungs of the nontreated mice (*p* < 0.01). Continuing the treatment after the initial prophylactic dose (P + T) reduced viral titers by 18.6 times (*p* < 0.01), resulting in the complete eradication of viruses from the lungs in 75% of the animals. A similar pattern was observed in the mice in the therapeutic treatment (TT) group at 24 h post‐infection, with the viral load reduced by 9.9 times (*p* < 0.01), and 62.5% of the animals presented no detectable viral load in their lung tissues. A previous study demonstrated the prophylactic potential of gold nanoparticles functionalized with sulfonated alkyl chains when they were administered intranasally prior to inoculation with a luciferase‐containing RSV.^[^ ^28^ ^]^ In contrast, our study demonstrated both the prophylactic and therapeutic efficacy of POSTAN using a relevant animal model and the wild‐type virus and measured viral infectivity through RT‒qPCR.

Next, we investigated the impact of POSTAN treatment on the lung pathology of infected animals. The acute phase of RSV‐A infection can be characterized by damage to the internal lining of alveoli (either type I or II pneumocytes) and endothelial cells, alveolar hemorrhage, interstitial and intra‐alveolar edema, thickening of the alveolar septum, and lymphocyte infiltration.^[^ ^76^, ^77^ ^]^ Figure 7a3 shows the lung tissue of untreated RSV‐A‐infected mice, which featured a thickened alveolar septum compared with that of healthy control mice (Figure 7a1). In addition to alveolar septum thickening, many red blood cells were observed around the alveolar septum in RSV‐infected tissues, as indicated by the black arrows. These cells were not present in healthy tissues. Neither lymphocyte infiltration nor hemorrhage was found in the infected lung tissues analyzed.

POSTAN treatment ameliorated the lung pathology of infected mice. The regions of thickened alveolar septum were reduced in the animals that received a single prophylactic dose of POSTAN (P group) (Figure 7c). Nonetheless, this reduction was not remarkable for all the animals, and red blood cells were still present in the tissues of these mice (Figure 7a4). In contrast, the P+T and TT groups experienced enhanced amelioration, marked by normal thickening of the alveolar septum (Figure 7c) and the absence of red blood cells along the alveolar interstices (Figure 7a5,a6).

## 3 Conclusion

We present the development of POSTAN, which effectively combats numerous HS‐dependent viruses. This compound has broad‐spectrum virucidal activity with an irreversible mechanism against a wide range of viral families. Through experiments conducted in cell lines, human‐derived pseudostratified and highly differentiated histocultures representing faithfully the human upper respiratory tract, and a relevant murine model of RSV‐A infection, we demonstrated the preventive and therapeutic efficacy of POSTAN. Additionally, the antiviral platform can serve as a nanocarrier for the delivery of other therapeutic molecules, as evidenced by internalization studies. In addition to their therapeutic applications, these virucidal nanoparticles could also be used for sterilizing and disinfecting a diverse range of surfaces, both living and nonliving. As such, virucidal POSTAN represents a multifaceted solution poised to revolutionize our approach to viral infections, disinfection, and sterilization, offering a promising avenue for addressing critical challenges in healthcare and beyond.

## 4 Experimental Section

### Materials and Chemicals

Glyceryl dibehenate (Compritol ATO 888, Gattefossé, Saint‐Priest, France); medium‐chain triglycerides (MCT) from Lipoid (Ludwigshafen, Germany); polyoxyethylene sorbitan oleate (PO) (polysorbate 80), sodium taurodeoxycholate (ST), sodium lauryl sulfate (SLS), fluorescein 5(6)‐isothiocyanate, and glutaraldehyde solution (50%) from Merck (Darmstadt, Germany); Ribavirin from Ambeed (Buffalo Grove, IL, USA); hydroxychloroquine, paraformaldehyde (PFA), resazurin, Dulbecco's phosphate buffered saline (DPBS), stabilized antibiotic and antifungal solution, microcrystalline cellulose (MCC), agarose (AGR), d‐α‐tocopheryl polyethylene glycol succinate, and mucin from porcine stomach type III (Sigma‐Merck, Saint Louis, MO, USA); penicillin, streptomycin, fetal bovine serum (FBS), trypsin‐EDTA (0.25%), Dulbecco's modified Eagle's medium (DMEM), DMEM/F12, CyQuant LDH Cytotoxicity Assay Kit, TRIzol reagent, TaqMan Universal PCR Master Mix, SYBR Green Universal Master Mix, and high‐capacity cDNA reverse transcriptase from Thermo Fisher Scientific (Waltham, MA, USA); PneumaCult‐Ex Plus medium from StemCell Technologies (Vancouver, BC, Canada); and a Direct‐Q 3 UV Milli‐Q water purification system (Millipore, Burlington, MA, USA) were used to prepare the solutions.

### Antiviral Nanoparticle Preparation and Characterization

Melting homogenization coupled with sonication was used to prepare POSTAN. Briefly, the oily phase, comprising Compritol ATO 888 (1.2% w/w), MCT (0.3% w/w), and PO (1% w/w), and the aqueous phase, an ST solution in PBS (0.5% w/w), were heated to 85 °C for 1 h and mixed via a homogenizer at 9000 rpm for 2 min. The hot preemulsion was then sonicated (20 kHz, 20% amplitude for 10 min in continuous mode) and cooled to room temperature (∼23 °C).

For cytometry and confocal analysis, fluorescein 5(6)‐isothiocyanate (FITC) (0.1% m/v) was solubilized in the oily phase to obtain the fluorescently labeled POSTAN (F‐POSTAN). FITC dispersions at the same concentration were prepared in DMSO for cell studies.

For the mechanism of action studies, nanoparticles containing sodium 10‐undecene‐1‐sulfonate (CN1) or d‐α‐tocopheryl polyethylene glycol succinate (CN2) were prepared using the same method and composition as POSTAN, with ST substituted accordingly. A 0.50% m/v solution of sodium 10‐undecene‐1‐sulfonate‐grafted cyclodextrins (CD1)^[^ ^29^ ^]^ was prepared in PBS and used as a control.

### Antiviral Nanoparticle Preparation and Characterization—Physical‐Cochemical Characterization

The hydrodynamic diameter (HD) and polydispersity index (PDI) of the nanoparticles were determined via dynamic light scattering (DLS) measurements with a Malvern Zetasizer Nano ZS90 at a scattering angle of 90° at 25 °C (Malvern Panalytical, Malvern, UK), with size distribution analyzed in intensity mode. The zeta potential (ZP) was measured via laser Doppler microelectrophoresis with the same instrument after dilution in a 1 mmol L^−1^ KCl solution. The number of particles was determined via a NanoSight NS 300 nanoparticle tracker (Malvern Panalytical, Malvern, UK) and Nanoparticle Tracking Analysis (NTA) 3.1 software.

The percentage of FITC incorporated into F‐POSTAN was assessed via the ultrafiltration method^[^ ^62^, ^78^ ^]^ with Amicon Ultra 4 filters (50 000 Da; Merck Millipore, Burlington, MA, USA) at 3000 × g for 15 min. The aqueous filtrate containing free FITC was analyzed by UV spectrophotometry (Shimadzu, Kyoto, Japan) at 470 nm after dilution with MeOH/H_2_O (80:20 v/v). A calibration curve with known FITC concentrations in MeOH/H_2_O (80:20 v/v) was used to determine the concentration of nonencapsulated FITC. The method was validated, showing linearity between 20 and 60 µg mL^−1^ (y = 0.017x – 0.017; r = 0.9941) and acceptable accuracy and precision rates ranging from 91–108% and 0–5%, respectively.^[^ ^79^ ^]^ The detection and quantification limits were 1 and 4 µg mL^−1^, respectively. The percentage of FITC incorporated was calculated by subtracting the free FITC from the total FITC added during nanoparticle preparation, dividing by the total FITC added, and multiplying by 100.^[^ ^62^ ^]^

### Antiviral Nanoparticle Preparation and Characterization—Morphology

POSTAN size and shape were evaluated via transmission electron microscopy (TEM) and cryogenic electron microscopy (cryo‐EM). For TEM, the nanoparticles were diluted with double‐distilled water at a 1:100 dilution and deposited onto a lacey carbon‐coated copper grid using 2% m/v uranyl acetate as a contrast agent. Image acquisition was performed via a JEM100CX2 microscope (JEOL, Tokyo, Japan) with an accelerating voltage of 100 kV. For cryo‐EM images, the samples were vitrified via a freeze plunger, and the data were acquired via a Tecnai F20 microscope (FEI, Hillsboro, OR, USA) equipped with a direct electron detection camera. TEM images from several fields were acquired and analyzed in ImageJ software for particle diameter measurement. The measurement tool was calibrated from pixels to micrometers using the original image scale.

### Antiviral Nanoparticle Preparation and Characterization—Thermal Analysis

Differential scanning calorimetry (DSC) thermograms were obtained for the Compritol ATO 888, PO, MCT, ST, and POSTAN formulations via a thermal analysis calorimeter (Perkin Elmer, Waltham, MA, USA). After freeze‐drying, each sample (3 mg) was hermetically sealed in aluminum pans. The samples were subsequently heated from 20 to 350 °C at a rate of 10 °C min^−1^. Baselines were determined via an empty pan, and all the thermograms were baseline‐corrected. The transition temperatures were determined from the endothermic peak minima, whereas the transition enthalpies were acquired by integrating the endothermic transitions via linear baselines.

### Antiviral Nanoparticle Preparation and Characterization—Release Study

The release profile of FITC from F‐POSTAN was assessed in PBS at pH 7.4 supplemented with 0.75% w/v sodium lauryl sulfate (SLS) to ensure sink conditions. Dialysis experiments were conducted using cellulose membrane bags (molecular weight cut‐off of 3500 Da) containing F‐POSTAN (2 mL; 20 mg of FITC). The bags were immersed in SLS/PBS dissolution medium (20 mL) and maintained under magnetic stirring at 25 °C. At predetermined time points, aliquots (1 mL) were withdrawn and replaced with an equal volume of fresh medium. FITC release was quantified via a UV‐1800 spectrophotometer (Shimadzu, Kyoto, Japan) at 470 nm following sample dilution in SLS/PBS. A validated calibration curve in SLS/PBS confirmed the method's linearity, precision, and accuracy within the 20–1000 µg mL^−1^ range (y = 0.007× + 0.02; r = 0.9979).^[^ ^79^ ^]^ Additionally, the physicochemical stability of F‐POSTAN in SLS/PBS was evaluated by measuring the HD, PDI, and ZP.

### Antiviral Nanoparticle Preparation and Characterization—Nebulization Studies

The colloidal stability of POSTAN after nebulization was evaluated via an air‐jet nebulizer (Soniclear, São Paulo, Brazil) and a homemade twin‐stage impinger (TSI) divided into three compartments representing the upper (S0 and S1) and lower (S2) airways (Figure S3, Supporting Information). According to the nebulizer's manufacturer, the nebulizer operates at a pressure of 30 psi generated by a mechanical piston, with an airflow of 10 L min^−1^ and a nebulization rate of 0.3 mL min^−1^ (without liquid), resulting in 50% droplets smaller than 5 µm. The airflow through the TSI system was drawn via a vacuum pump (Millipore, Burlington, MA, USA), with the airflow rate set at –7.5 cm H_2_O (simulating the negative pressure in standard inspiration patterns), and it was continuously monitored during the experiments. POSTAN (2 mL) was nebulized for 8 min.

A 1 mmol L^−1^ KCl solution was used as the collection liquid in S1 (10 mL) and S2 (20 mL) to analyze the physicochemical properties of POSTAN deposited in the TSI stages. Samples (1 mL) were directly analyzed to determine the HD, PDI, and ZP values. Nanoparticles in S0 were collected and analyzed after washing the stage with 0.1 mmol L^−1^ KCl solution (1 mL).^[^ ^52^, ^53^, ^63^ ^]^

### Antiviral Nanoparticle Preparation and Characterization—Mucin Interaction

The absorbance and physicochemical properties of the nanoparticles after incubation with a mucin dispersion were analyzed.^[^ ^52^, ^53^, ^80^, ^81^ ^]^ First, a mucin dispersion was prepared at a concentration of 0.08% w/v in PBS, stirred overnight, and then centrifuged at 8000 × g for 20 min at 4 °C to obtain the mucin‐containing supernatant. This mucin mixture was then mixed with POSTAN at a 1:1 volume ratio and vortexed for 1 min. The absorbance of the mixture was recorded at 650 nm initially and after 30, 60, and 120 min of incubation at 37 °C and 100% relative humidity to model the conditions of the airways. The absorbance of the mucin dispersion and POSTAN at 650 nm was also measured as a reference. The interaction between mucin and POSTAN was further examined by evaluating the HD, PDI, and ZP of the mucin‒nanoparticle mixture, as described previously.

### In Vitro Cell Studies—Cell Culture

African green monkey kidney epithelial cells (Vero CCL81, ATCC Manassas, VA, USA) were cultured in DMEM supplemented with 10% v/v FBS and 1% v/v antibiotic/antifungal mixture containing penicillin (10 000 IU mL^−1^), streptomycin (10 mg mL^−1^), and amphotericin B (25 µg mL^−1^). The cells were maintained at 37 °C in a 5% CO_2_ atmosphere.

### In Vitro Cell Studies—Cytotoxicity Assays

POSTAN cytotoxicity was assessed via two different protocols: first, the cells were exposed to the nanoparticles diluted in culture medium for 3 or 24 h; second, the cells were exposed to the nanoparticles incorporated into a gel overlay of 0.5% w/v carboxymethylcellulose (CMC) containing DMEM plus 3% v/v FBS (0.5% CMC‐DMEM‐3% FBS) for 48 h.

For the first experiment, cells (200 µL, 2 × 10^4^ cells per well) were seeded in 96‐well plates and incubated overnight. The culture medium was then removed, and the cells were rinsed with PBS before incubation for 3 or 24 h in serum‐free DMEM containing POSTAN (200 µL, 5.6 × 10^11^–11.6 × 10^2^ particles mL^−1^, which corresponded to 4000–0.6 µg mL^−1^ ST content).

For the gel‐containing POSTAN experiment, cells (500 µL, 1 × 10^5^ cells per well) were seeded in 24‐well plates. The next day, 0.5% CMC‐DMEM‐3% FBS containing POSTAN (500 µL, 1.6 × 10^11^–1.6 × 10^9^ particles mL^−1^, which corresponded to 200–2 µg mL^−1^ ST content) was added to the cells, which were subsequently incubated for 48 h. A dispersion of 0.5% CMC in DMEM plus 3% FBS was used as a control. The cells were rinsed with PBS, and viability was assessed via a resazurin assay. A resazurin solution in serum‐free DMEM (200 µL, 15 µg mL^−1^) was added to each well. After a 4‐h incubation, the resorufin fluorescence was measured at excitation and emission wavelengths of 530 and 590 nm, respectively, via a Synergy HTX microplate reader (Biotek, Winooski, VT, USA). Cell viability (%) was determined by dividing the fluorescence signal of the treated groups by that of the untreated groups.

### In Vitro Cell Studies—Cellular Internalization Assays

POSTAN cellular internalization studies were performed with F‐POSTAN in Vero CCL81 cells via flow cytometry and confocal laser scanning microscopy (CLSM).

For flow cytometry, cells were seeded into 96‐well plates (8 × 10^4^ cells per well) in complete DMEM and incubated for 24 h at 37 °C. The cells were washed with PBS and incubated in FBS‐free DMEM containing FITC solution or F‐POSTAN (200 µL, 8 × 10^10^ particles mL^−1^) for 30 min. Following the washing step, trypsin (50 µL, 0.25% v/v) was added for 5 min at 37 °C within a 5% CO_2_ atmosphere to facilitate cell detachment. Trypsin was subsequently neutralized by the addition of complete DMEM (50 µL), and the samples were analyzed using a Guava EastCyte 8HT flow cytometer (Millipore, Burlington, MA, US) after the addition of propidium iodide (PI) (2 µL, 50 µg mL^−1^ in PBS).

For CLSM, cells were seeded into 6‐well plates containing sterilized coverslips (22 mm × 22 mm) (10^5^ cells per well) in complete DMEM. The next day, the cells were washed and incubated in FBS‐free DMEM containing POSTAN (1 mL, 8 × 10^10^ particles mL^−1^), F‐POSTAN (1 mL, 8 × 10^10^ particles mL^−1^; 0.1% w/v FITC), and 0.1% m/v FITC solution (1 mL) for 30 min. POSTAN was utilized as a control for the absence of basal fluorescence from the formulation. The cells were washed with PBS and fixed with 4% w/v PFA. The coverslips were then mounted with Fluoromount mounting medium containing DAPI before visualization via CLSM. The samples were observed via a TCS SP8 scanning confocal microscope (Leica, Wetzlar, Germany) with a 63× immersion objective.

### Antiviral Mechanism of Action

The Vero CCL81, Vero E6, and Calu‐3 cell lines were obtained from ATCC (Manassas, VA, USA).

HSV‐2 (a clinical strain isolated at the University Hospital of Geneva) was propagated, collected 3 days post infection (dpi), clarified, aliquoted, frozen at −80 °C, and titrated via a plaque assay in Vero E6 cells. ZIKV (BeH819966 strain), RSV‐A (long strain), and CHIKV (S27 African strain), obtained from the Virology Research Center, School of Medicine of Ribeirão Preto, University of São Paulo, were propagated, collected at 7 dpi, clarified, aliquoted, frozen at −80 °C, and subsequently titrated via plaque assays in Vero CCL81 cells.

SARS‐CoV‐2 (omicron/B.1.1.529 strain, kindly provided by the Centre for Emerging Viral Diseases in Geneva) was propagated on Calu‐3 cells, collected at 3 dpi, aliquoted, frozen at −80 °C, and subsequently titrated via a plaque assay in Vero E6 cells.

### Antiviral Mechanism of Action—Inhibition Assays

Vero CCL81 cells were seeded in 24‐well plates (10^5^ cells per well) in complete DMEM and incubated at 37 °C and 5% CO_2_ for 24 h. Different durations of antiviral administration were subsequently tested; for each condition, a vehicle‐treated control was included.

a) Cell pretreatment assay: Cells were treated for 3 h at 37 °C with increasing concentrations of POSTAN. The formulation was removed, and the cells were washed with PBS and then inoculated with HSV‐2 (200 µL; 10^3^ PFU mL^−1^; MOI 0.002) for 1 h at 37 °C. The inoculum was aspirated, and the cells were overlaid with 0.5% CMC in DMEM plus 3% FBS and incubated at 37 °C and 5% CO_2_ for 48 h.

b) Virus pretreatment: HSV‐2 (200 µL; 1 × 10^4^ PFU mL^−1^; MOI 0.02), SARS‐CoV‐2 (Delta: 200 µL; 2 × 10^4^ PFU mL^−1^; MOI 0.01; Omicron: 200 µL; 4 × 10^4^ PFU mL^−1^; MOI 0.01), VSV‐▵‐CoV‐2 (MOI 0.1), RSV‐A (200 µL; 5.5 × 10^5^ PFU mL^−1^; MOI 0.1), ZIKV (200 µL; 5 × 10^3^ PFU mL^−1^; MOI 0.005), and CHIKV (200 µL; 4 × 10^4^ PFU mL^−1^; MOI 0.01) were incubated with increasing concentrations of POSTAN, CN1, CN2 or ribavirin for 2 h at 37 °C, after which this mixture was added to the cells for 1 h at 37 °C. The following overlay was applied for the respective assays and incubated at 37 °C with 5% CO2:

– For HSV‐2, 0.5% CMC in DMEM with 3% FBS was added, and the mixture was incubated for 2 days.
– For SARS‐CoV‐2, 2.5% methylcellulose (MMC) in DMEM with 5% FBS was added, and the mixture was incubated for 3 days.
– For RSV‐A, 0.4% agarose in DMEM with 2% FBS was added, and the mixture was incubated for 6 days.
– For ZIKV, the cells were incubated with 2.5% CMC in DMEM supplemented with 5% FBS for 7 days.
– For CHIKV, the cells were incubated with 1.5% CMC in DMEM supplemented with 3% FBS for 3 days.

c) Postinfection treatment: Cells were infected with HSV‐2 (200 µL; 1 × 10^3^ PFU mL^−1^; MOI 0.002) for 1 h at 37 °C. The cells were washed, overlaid with 0.5% CMC in DMEM supplemented with 3% FBS containing increasing concentrations of POSTAN, and incubated at 37 °C and 5% CO_2_ for 48 h.

After the incubation period indicated for each virus, a plaque assay was performed. The overlay was aspirated, and the cells were fixed with 4% w/v PFA for 1 h, followed by staining with 0.1% w/v crystal violet in a 20% v/v ethanol solution for 1 h under gentle shaking. Plaques were counted, and the percentage of viral infectivity inhibition was determined by comparing the number of plaques in the treated versus untreated wells. The selectivity index (SI), defined as the ratio of the CC_50_ to the IC_50_, was employed to assess the possibility of employing POSTAN as an antiviral.^[^ ^82^ ^]^

### Antiviral Mechanism of Action—Virus Penetration Assay

The penetration assay was performed under two distinct conditions: warm binding conditions (37 °C), which permits virus attachment and subsequent entry, and cold binding conditions (4 °C), where virus attachment to the cell membrane occurs, while internalization was hampered.^[^ ^37^ ^]^

Vero CCL81 cells were seeded in 24‐well plates (10^5^ cells per well) in complete DMEM and incubated at 37 °C for 24 h. The cells were inoculated with HSV‐2 (200 µL; 10^3^ PFU mL^−1^; MOI 0.002) for 45 min at 37 °C in a warm assay. The virus was removed, and the cells were washed with warm serum‐free DMEM at 37 °C to remove excess virus. For the cold binding assay, the cells were chilled in an ice bucket for 30 min, inoculated with cooled HSV‐2 (200 µL; 10^3^ PFU mL^−1^; MOI 0.002), and incubated for 45 min at 4 °C. The viral inoculum was then aspirated, and the cells were washed with cooled serum‐free DMEM to remove unbound virus from the cell membrane.

Subsequent to virus inoculation, 200 µL of POSTAN at 100 µg mL^−1^ (based on the ST content) diluted in serum‐free DMEM was added to the cells for 2 h in both assays. The cells were washed with PBS, overlaid with 0.5% CMC in DMEM plus 3% FBS and incubated at 37 °C and 5% CO_2_ for 48 h. The plaque assay was then performed as outlined previously. The percentage of virus infectivity inhibition was determined by comparing the number of plaques in treated wells to that in untreated control wells. Notably, equivalent viral titers were used for both the cold (4 °C) and warm (37 °C) penetration assays, and the same number of plaques were observed in the untreated controls. CD1 was used as a positive control.^[^ ^29^ ^]^

### Antiviral Mechanism of Action—Virucidal Assay

The principle of this test was as follows: if an antiviral compound acts extracellularly with virustatic properties, diluting the virus‒compound complex will restore viral infectivity, enabling plaque counting. Conversely, if the antiviral compound has virucidal properties, the virus will lose its ability to infect cells upon dilution, leading to either the absence of detectable plaques or a reduced plaque count compared with the control.^[^ ^28^, ^29^ ^]^ HSV‐2 (100 µL; 1 × 10^5^ PFU mL^−1^; MOI 0.1), SARS‐CoV‐2 (Delta: 100 µL, 1 × 10^6^ PFU mL^−1^; MOI 1; Omicron: 100 µL; 1 × 10^7^ PFU mL^−1^; MOI 10), RSV‐A (100 µL; 1 × 10^5^ PFU mL^−1^; MOI 0.1), ZIKV (90 µL; 1 × 10^4^ PFU mL^−1^; MOI 0.01), and CHIKV (90 µL; 1 × 10^4^ PFU mL^−1^; MOI 0.01) were incubated with POSTAN at 100 µg mL^−1^ (based on the ST content) or serum‐free DMEM for 2 h at 37 °C. Serial dilutions of this mixture were performed in serum‐free DMEM and then added to the cells for 1 h at 37 °C. The cells were washed with PBS, and the virus‐specific overlay and incubation periods at 37 °C and 5% CO_2_ were applied to each virus as described previously. Viral titers were determined via plaque assays upon serial dilutions of the samples until the nanoparticles lost effectiveness (concentration below their EC_50_). The results were compared with those from untreated control wells. The same assay was performed with CD1, CN1, and CN2 formulations. CD1 was used as a positive control.^[^ ^29^ ^]^ CN1 and CN2 were used as a negative control due to their virustatic activity.

### Antiviral Mechanism of Action—DNA Exposure Assay

To evaluate the degree of virus damage induced by virus exposure with POSTAN, a DNA exposure and amplification assay was performed. HSV‐2 (30 µL; 1 × 10^5^ PFU mL^−1^) was incubated for 2 h at 37 °C with DMEM or POSTAN at 100 µg mL^−1^ (based on the ST content). The mixtures were subsequently diluted at a 1:20 ratio in serum‐free DMEM, and 100 µL of the resulting dilutions were either exposed to 8 units of Turbo DNase or incubated with only Turbo DNase buffer for 30 min at 37 °C. At the end of the incubation, the samples were subjected to DNA extraction through NucliSENS EasyMag. Finally, qPCR amplification was performed with TaqMan Universal PCR Master Mix with the following primers and probes: primers (for 5′‐CCGTCAGCACCTTCATCGA ‐3′ and Rev 5′‐CGCTGGACCTCCGTGTAGTC ‐3′); probes (5′‐FAM CCACGAGATCAAGGACAGCGGCC TAMRA). The viral DNA extracted from the HSV‐2 viral stock was used as a reference standard. The fold change was calculated via the delta Ct method. CD1 was used as a positive control.^[^ ^29^ ^]^

### Antiviral Mechanism of Action—Transmission Electron Microscopy (TEM)

Concentrated HSV‐2 viral stocks were prepared to visualize POSTAN‐induced damage to viral particles via TEM. Briefly, three Petri dishes were infected for 2 days, and the supernatants were collected and centrifuged at 3000 × g for 15 min at 4 °C to sediment the cell debris. The clarified supernatant was then transferred to Ultra‐Clear ultracentrifugation tubes (Beckman Coulter, Brea, CA, USA) and subjected to ultracentrifugation via an Optima L‐70K ultracentrifuge (Beckman Coulter, Brea, CA, USA) at 30,000 × g for 2 h at 4 °C to sediment the viral particles. The supernatant was removed, and virions were resuspended in nonsupplemented DMEM (100 µL). The viral titer determined by the plaque assay was 4.5 × 10^9^ PFU mL^−1^. For the POSTAN‐exposed virus, the viral stock (10 µL) was mixed with POSTAN (10 µL; 100 µg mL^−1^ on the basis of the ST content) and incubated for 2 h at 37 °C. The sample was then fixed with 4% v/v glutaraldehyde (40 µL). For TEM imaging, the fixed samples (5 µL) were adsorbed onto carbon grids and negatively stained with 0.5% uranyl acetate for observation. Unexposed HSV‐2 and POSTAN alone were used as controls.

### Ex Vivo Studies—Establishment of Human Airway Epithelial (HAE) Tissue Culture

The 3D histocultures were derived from human bronchial biopsies of patients with noninfectious or noncancerous lung disease admitted to the Hôpitaux Universitaires de Genève (HUG) (Ethics Committee Project Number: PB 2021‐00042 14 062). Briefly, bronchial epithelial cells were mechanically dissociated from the biopsies and resuspended in PneumaCult‐Ex Plus medium (StemCell Technologies, Vancouver, Canada) in 6‐well plates coated with fibronectin and collagen until 70–80% confluence. The cells were then expanded in a culture flask until they reached confluence and subsequently plated (1.1 × 10^5^ cells per well) on 12‐well Transwell plates (0.4 µm pore size, 1.12 cm^2^ surface area; Costar, Corning, NY, USA) in both the apical and basal compartments with PneumaCult‐Ex Plus medium. Once the cell confluence reached 90–100%, the tissues were placed at the air‒liquid interface (ALI) by removing the apical medium and were cultured with PneumaCult ALI differentiation medium (StemCell Technologies, Vancouver, Canada) in the basal compartment. The differentiation medium was replaced every two days for 21–28 days to achieve differentiation of mucus and basal cell populations and a reconstituted respiratory epithelium mimicking the pseudostratified architecture of the human airway epithelium.

Several parameters, including cilia beating frequency, mucus secretion, tissue metabolic activity, and transepithelial electrical resistance (TEER), were assessed to characterize and verify the physiology and integrity of the tissues. The presence of basal, ciliated, mucus‐secreting cells and tight junctions was confirmed by immunostaining different cell populations with specific markers. For all the experiments, epithelia were prepared from three different single‐donor biopsies. All inserts were sterile and tested negative for HIV1, mycoplasma, hepatitis B, hepatitis C, bacteria, yeast, and fungi. They presented TEER values above 200 Ω.cm^2^ (278–553 Ω.cm^2^), cilia beating frequencies above 5 Hz (8.3–9.1 Hz), and a normal morphology with a mucus layer or some mucus vesicles.

### Ex Vivo Studies—Toxicity Assays

POSTAN toxicity was assessed in HAE tissue cultures via resazurin and lactate dehydrogenase (LDH) assays. POSTAN (30 µL; concentration range of 0.4 × 10^12^–0.8 × 10^13^ particles mL^−1^ or 0.5–10 mg mL^−1^ on the basis of the ST content) was applied daily on the apical side of the tissues.

For the resazurin assay, the basal medium was replaced with fresh medium containing resazurin (500 µL; 15 µg mL^−1^), and the mixture was incubated for 4 h at 37 °C. The fluorescence was recorded via 560‐nm excitation and 590‐nm emission filter sets and compared with that of untreated tissues. Before POSTAN administration, the tissues were rinsed three times with PBS in both the apical and basal compartments, and fresh culture medium (500 µL) was added to the basal compartments.

For the LDH assay, POSTAN (30 µL; 0.4 × 10^12^ particles mL^−1^; 0.5 mg mL^−1^ on the basis of the ST content) was applied daily on the apical side of the tissues. At the 24‐h time point, the formulation was aspirated, and fresh molecules were reapplied to the apical compartment. The basal medium was collected daily, stored at −20 °C, and replaced with fresh culture medium (500 µL). LDH released into the basal medium was measured with a CyQuant LDH Cytotoxicity Assay Kit following the manufacturer's instructions.

### Ex Vivo Studies—Preventive and Therapeutic Efficacy Assays

For the prophylactic assay, tissues were treated on the apical surface with either POSTAN (30 µL; 0.4 × 10^11^ particles mL^−1^; 0.5 mg mL^−1^ on the basis of the ST content) or REGEN‐COV, a combination of casirivimab and imdevimab (30 µL, with each antibody at 600 mg), 30 min prior to SARS‐CoV‐2 inoculation (100 µL; 4 × 10^4^ PFU mL^−1^). For the therapeutic treatment assay, POSTAN or REGEN‐COV was added to the tissues 18 h post‐infection (hpi) at the same concentrations and conditions as above. The compounds were diluted in 30 µL of culture medium to enable administration while maintaining a viable microenvironment, allowing virions to be released from the tissue for collection and subsequent analysis.

Following virus inoculation at 37 °C and 5% CO_2_ for 4 h, the tissues were washed three times with culture medium to remove excess virus. The cultures were then maintained at the air‒liquid interface with fresh culture medium (500 µL) in the basal compartment. Eighteen hours postinfection, culture medium (200 µL) was applied to the apical compartment for sample collection by gentle pipetting, and fresh POSTAN was reapplied to the apical compartment. The basal medium was collected and replaced with fresh medium (500 µL). This procedure was repeated daily for 3 days.

Viral RNA extracted from the apical washes of the HAE tissue cultures was quantified via RT‒qPCR. SARS‐CoV‐2 RNA copies were quantified via the standard reference transcript of the SARS‐CoV‐2 E gene as described previously.^[^ ^83^, ^84^ ^]^ Ct values were converted into RNA loads via the slope‐intercept method.^[^ ^83^ ^]^

### In Vivo Studies—Animals

C57BL/6 mice were obtained from the Animal Facility of the University of São Paulo (Ribeirão Preto Campus) and transported to the Animal Facility of the Virology Research Center. The number of animals per cage was 9, consisting of 1 adult mother (8 weeks old and 25 g) and eight 2‐day‐old newborns weighing 1–2 g. Food and water were provided *ad libitum* to the adult animals. Newborn mice were breastfed throughout the experiment. This study was conducted in accordance with the Ethics Committee on the Use of Animals (CEUA) of the School of Medicine of Ribeirão Preto, University of São Paulo, and in compliance with the respective Brazilian Federal and State Laws 11 794 of 10/08/2008 and 11 977 of 08/25/2005 under authorization number 1273/2023R1. All the experimental procedures were performed in a Biosafety Level 2 facility of the Virology Research Center, School of Medicine of Ribeirão Preto at the University of São Paulo.

### In Vivo Studies—Safety in Healthy Mice

To evaluate the potential toxicity of POSTAN upon daily intranasal administration, the nanocarriers (10 µL) or PBS (10 µL) were administered to healthy neonate mice intranasally (IN) once per day for 4 days. On the 5th day, the animals were euthanized for sample collection, and the lung tissues were dissected, washed with PBS, and immediately fixed in 10% v/v PFA overnight. To analyze potential tissue damage and inflammation following POSTAN administration, the samples were dehydrated through a graded alcohol series, embedded in paraffin, and cut into 5 µm sections. Hematoxylin and eosin (HE) staining was performed on these sections. The slides were then examined via light microscopy with the assistance of a pathologist.

### In Vivo Studies—Efficacy in RSV‐A‐Infected Mice

Ten microliters (10 µL) of the RSV‐A stock (850 PFU per gram of animal) or 10 µL of the formulations (5 µL per nostril) was administered intranasally under anesthesia (1.0–2.0% v/v isoflurane) following established protocols.^[^ ^68^ ^]^ Animals were separated into the following groups: a) Control group: PBS was administered once daily (*n* = 8); b) RSV group: PBS was administered once daily starting at 24 hpi (*n* = 12); c) Single prophylaxis dose group (P group): A single dose of POSTAN was administered 15‒25 min before viral infection, followed by daily PBS administration (*n* = 8); d) Prophylaxis and treatment group (P+T group): POSTAN was administered 15‒25 min before viral infection, followed by repeated administrations once per day for 4 days, starting at 24 hpi (*n* = 8); e) Therapeutic treatment group (TT group): POSTAN was administered once per day for 4 days, starting at 24 hpi (*n* = 8). POSTAN was administered intranasally at a concentration of 5 mg mL^−1^ on the basis of the ST content, corresponding to 4.3 × 10^12^ particles mL^−1^.

The animals were monitored every morning for 4 days to assess weight gain and survival after infection. On the 5th day, the animals were euthanized, and samples were collected for viral titer quantification and histological analysis.

Viral titers in the lung tissues were quantified via RT‒qPCR as previously described.^[^ ^85^ ^]^ Briefly, lung tissues were dissected, washed with PBS, and weighed, and RNA was extracted with TRIzol according to the manufacturer's instructions via the Tissue Lyser LT (Qiagen, Hilden, Germany). Five hundred nanograms of total RNA per sample and SYBR green PCR master mix were used (primers: forward, ACA ACA AAC TTG CGT AAA CCA AAA; reverse, CCA TGC TAC TTC ATC ATT GTC AAA CA) for the HRSV leader region and (forward, GCTCTTAGCAAAGTCAAGTTGAATGA; reverse, TGCTCCGTTGCATGGTGTATT) for the hRSV N gene.^[^ ^85^ ^]^ The viral RNA extracted from the RSV‐A viral stock was used as a reference standard. Ct values were converted into RNA copies per sample via the slope‐intercept method.

To assess the effects of POSTAN treatment on the lung pathology of RSV‐infected mice, lung tissues were processed as previously described. HE staining was performed on these sections. The slides were then examined via light microscopy with the assistance of a pathologist. Thickening of the alveolar septum was measured from histological images acquired via light microscopy. Measurements were performed via ImageJ. The measurement tool was calibrated from pixels to micrometers on the basis of the original scale image.

### Statistical Analysis

Independent replicates of in vitro and ex vivo experiments (*n* = 3–6) and in vivo experiments (*n* = 8–16) were performed. Prior to statistical testing, data were log‐transformed when appropriate or deemed advantageous for analysis. Specifically, nanoparticle concentrations in cell viability and in vivo antiviral studies, as well as viral titers in ex vivo and in vivo antiviral studies, underwent logarithmic transformation. Data distribution was assessed using the Shapiro–Wilk test. Results were presented as mean ± standard deviation (SD). When normality assumptions were met, comparisons between two groups were performed using a two‐tailed Student's *t* test. For multiple group comparisons, one‐way ANOVA followed by Tukey's or Dunnett's post hoc test, or two‐way ANOVA followed by Tukey's post hoc test, was applied. For non‐normally distributed data, the Mann–Whitney test (two groups) or the Kruskal–Wallis test followed by Dunn's post hoc test (multiple groups) was used. A significance level of *p* < 0.05 was applied for parametric tests and *p* < 0.01 for non‐parametric tests. All statistical analyses as well as the half‐maximal cytotoxic concentration (CC_50_)and half‐maximal inhibitory concentration (IC_50_) were calculated using Prism 7 (GraphPad Software, La Jolla, CA, USA).

### Ethics Approval Statement

All animal procedures were approved by the Ethics Committee on the Use of Animals (CEUA) of the School of Medicine of Ribeirão Preto, University of São Paulo, under protocol number 1273/2023R1 and conducted in accordance with relevant guidelines and regulations. Human biopsy collection was approved by the Commission cantonale d'éthique de la recherche (CCER ‐ Genève), under protocol number PB 2021‐00042 14 062.

### Patient Consent Statement

Informed consent was obtained from all human participants included in the study.

## Conflict of Interest

The authors declare no conflict of interest.

## Author Contributions

Y.A.M. conceptualized, designed, and managed the project and study, conducted experiments, analyzed the data, and wrote and revised the manuscript with input from supervisors. L.B. cultured the HAE tissues and assisted with the SARS‐CoV‐2 RT‐PCR analysis. A.C.Z. assisted with the HSV‐2 TEM analysis. T.M.L. performed the RSV‐A plaque assays with ribavirin and assisted with the animal experimentation. J.P.S. assisted with RSV‐A RT‐PCR analysis and animal experimentation. H.K.T. produced the enterovirus strains. E.A.N. provided the ZIKV (BeH819966 strain), RSV‐A (long strain), and CHIKV (S27 African strain) viruses, oversaw animal study authorization, and supplied research resources for in vivo antiviral studies. F.C. performed the pathological analysis of lung tissues. F.S. provided research resources. R.F.V.L. supervised the study and provided resources for nanoparticle development and characterization. C.T. supervised the study, designed the project, provided resources for in vitro and ex vivo antiviral studies, revised the manuscript, and oversaw authorization for human biopsy collection. All authors contributed to the discussion of the results. All images were created by the authors using BioRender.

## Supporting information



Supporting Information

## Data Availability

The data that support the findings of this study are available from the corresponding author upon reasonable request.
