# Supplementary material for: Novel Wide‐Spectrum Virucidal Lipid Nanoparticles
Source: Small. 2025 Nov 14;22(7):e07669. doi: 10.1002/smll.202507669 (PMC12862453; doi:10.1002/smll.202507669)
Supplement: Supplementary file 1 — Supporting Information [file SMLL-22-e07669-s001.docx]

**Novel Wide-Spectrum Virucidal Lipid Nanoparticles**

Yugo Araújo Martins^1,2,5^, Louise Bondeelle^1^, Arnaud Charles-Antoine Zwygart^1^, Thais Melquiades de Lima^3^, Juliano de Paula Souza^3^, Han Kang Tee^2^, Fernando Chahud^4^, Eurico de Arruda Neto^3^, Francesco Stellacci^5^, Renata Fonseca Vianna Lopez^2^, Caroline Tapparel*^1^

^1^Department of Microbiology and Molecular Medicine, Faculty of Medicine, University of Geneva, Geneva 1211, Switzerland

^2^Department of Pharmaceutical Sciences, School of Pharmaceutical Sciences of Ribeirão Preto, University of São Paulo, Ribeirão Preto, SP 14040-900, Brazil

^3^Department of Molecular and Cell Biology, School of Medicine of Ribeirão Preto, University of São Paulo, Ribeirão Preto, SP 14040-900, Brazil

^4^ Department of Pathology, School of Medicine of Ribeirão Preto, University of São Paulo, Ribeirão Preto, SP 14040-900, Brazil

^5^Institute of Materials, École Polytechnique Fédérale de Lausanne, Lausanne 1015, Switzerland

**SUPPLEMENTARY MATERIAL**

**Table S1 - Physicochemical properties of POSTAN, FITC-labeled POSTAN (F-POSTAN) and control nanoparticles**

| **Parameter** |  | **POSTAN** | **F-POSTAN^#^** | **CN1**^&^ | **CN2**^&^ |
| --- | --- | --- | --- | --- | --- |
| **Hydrodynamic diameter (nm)** | DLS | 105 ± 8 | 95 ± 8 | 102 ± 17 | 97 ± 22 |
|  | NTA | 189 ± 51* | n/a | n/a | n/a |
|  | TEM^$^ | 135 ± 42 | n/a | n/a | n/a |
| **Polydispersity index** | | 0.15 ± 0.01 | 0.15 ± 0.04 | 0.11 ± 0.01 | 0.16 ± 0.02 |
| **Zeta potential (mV)** | | -22 ± 2 | -25 ± 1 | -16 ± 2* | -11 ± 1* |
| **Encapsulation efficiency (%)** | | n/a | 93 ± 1.5 | n/a | n/a |

Data are expressed as means ± SDs (n = 3-4); Statistical significance was calculated via Student’s t-test t with *p < 0.05 (DLS vs NTA or POSTAN vs CN1 or CN2); n/a : not applicable.

^$^TEM images from several fields were acquired and analyzed in ImageJ software for particle diameter measurement (n = 50).^#^F-POSTAN nanoparticles were prepared with 0.1% (m/v) FITC. ^&^In CN1, sodium taurodeoxycholate from POSTAN was replaced with sodium 10-undecene-1-sulfonate (a sulfonated, non-cholesterol compound), whereas in CN2 it was replaced with a tocopherol derivative (a non-sulfonated, non-cholesterol compound).

**
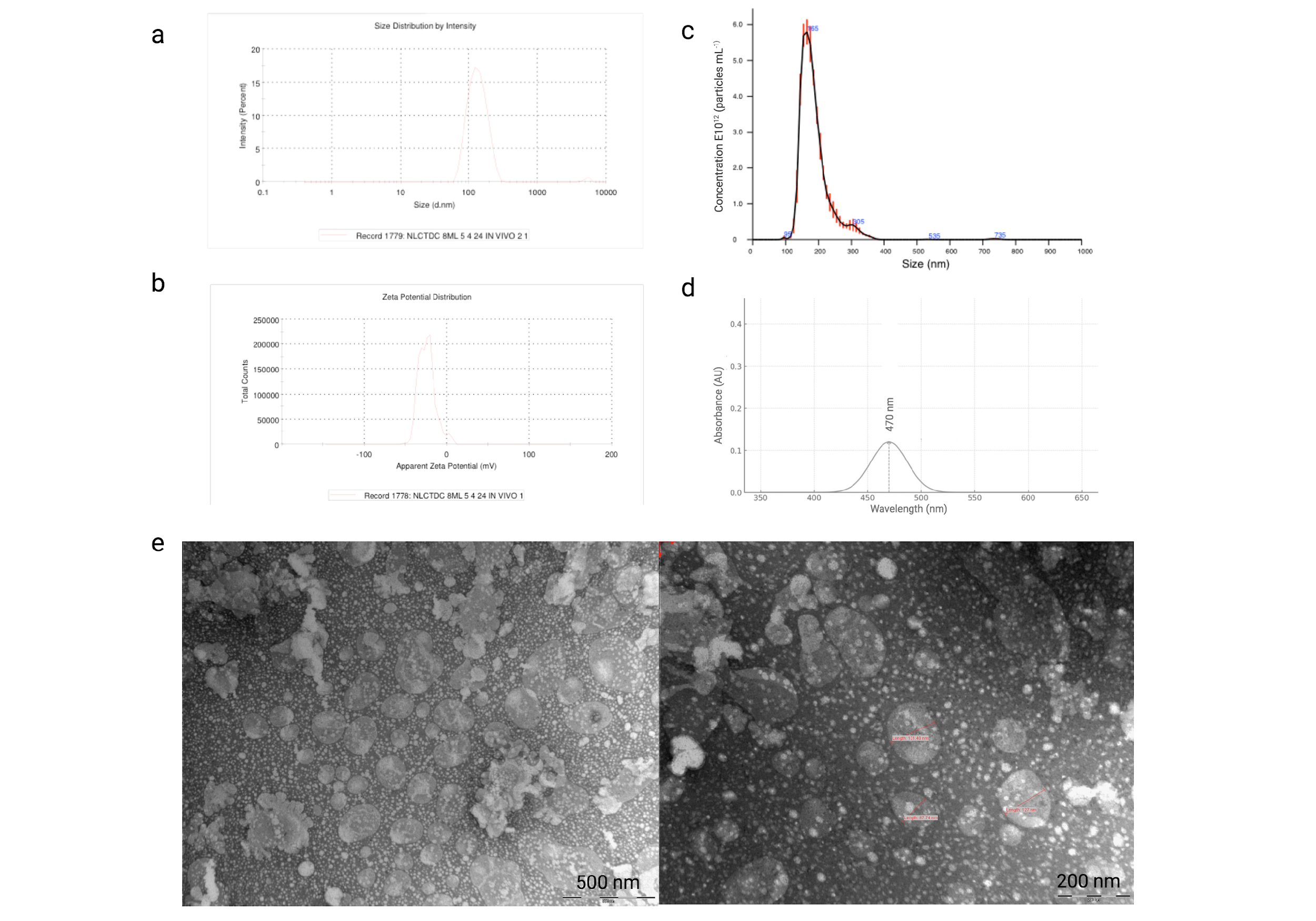
**

**Figure S1. Characterization of POSTAN nanoparticles.** (a) Hydrodynamic diameter determined by dynamic light scattering (DLS); (b) Zeta potential; (c) Nanoparticle tracking analysis (NTA); (d) UV–visible spectrum of free FITC; (e) Transmission electron microscopy (TEM) images.

**Figure S2. Release study.** FITC release profile from F-POSTAN over the 24-hour period, demonstrating FITC sustained release within the receptor medium (PBS) supplemented with 0.75% w/v sodium lauryl sulfate solution (SLS) (n = 3). To guarantee the sink conditions, 2 mL of F-POSTAN (corresponding to 2 mg of FITC) were placed in cellulose membrane dialysis bags of 3,500 Da molecular weight cut off and dialyzed against 20 mL PBS/SLS under magnetic stirring at room temperature. One milliliter of samples was collected from PBS/SLS at predetermined times and replaced with 1 mL of the same medium.

**Table S2 - Physicochemical properties of F-POSTAN before and after incubation in PBS/SLS**

| **Parameter** | **F-POSTAN before incubation in PBS/SLS** | **F-POSTAN after 24 incubation in PBS/SLS** |
| --- | --- | --- |
| **Hydrodynamic diameter (nm)** | 105 ± 8 | 119 ± 36 |
| **Polydispersity index** | 0.15 ± 0.01 | 0.15 ± 0.1 |
| **Zeta potential (mV)** | -22 ± 2 | -32 ± 3* |

Data are expressed as means ± SD (n=3); *Statistical significance was calculated via Student’s t-test t with p < 0.05;


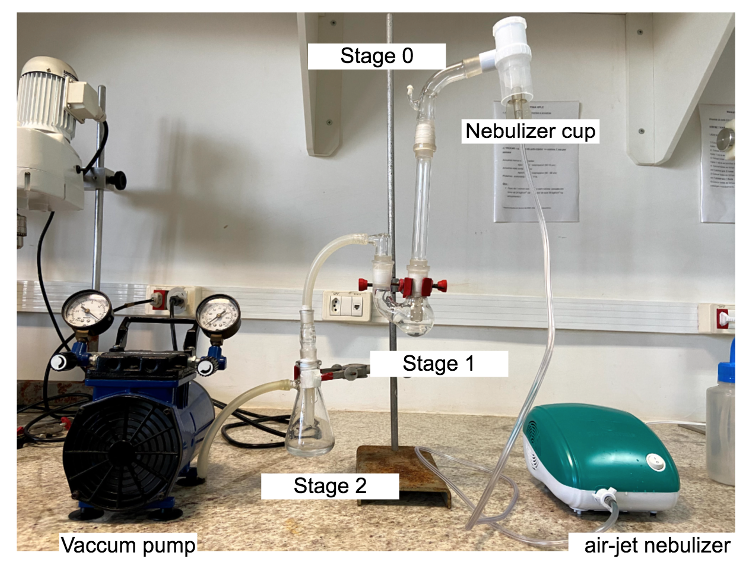


**Figure S3. Homemade TSI divided into three stages representing the upper (Stage 0 and Stage 1) and lower (Stage 2) stages of the airways.**


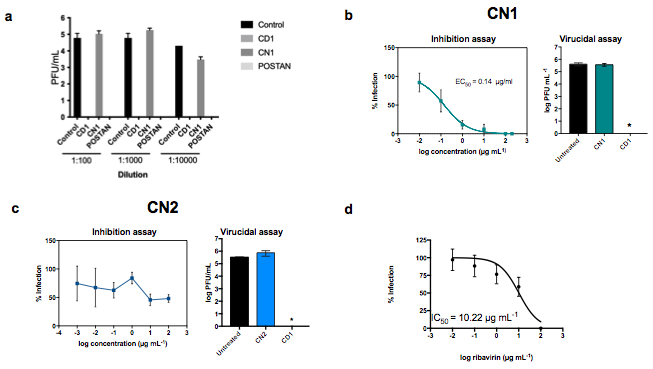


**Figure S4. Dose-response and virucidal assays for control nanoparticles and ribavirin.**
(a) Viral titers measured at different dilutions after incubation of HSV-2 with POSTAN, the virustatic compound CN1, and the virucidal compound CD1; (b) Dose-response and virucidal assays with CN1 and HSV-2; (c) Inhibition and virucidal assays with CN2 and HSV-2; (d) Dose-response assay with ribavirin and RSV-A. *Dose–response assays:* Serial dilutions of the nanoparticles were incubated with HSV-2 (MOI 0.02) or RSV-A (MOI 0.1) for 2 h at 37 °C prior to cell infection (virus pretreatment). HSV-2 and RSV-A inhibition and IC_50_values were respectively determined at 2 and 6 days post-infection by plaque assay and compared with untreated controls (n = 3–4). *Virucidal assays:* HSV-2 (MOI 0.1) was incubated with POSTAN, CD1, CN1, or CN2 for 2 h at 37 °C, followed by serial dilution and infection of Vero cells. Viral titers of untreated and treated HSV-2 were measured by plaque assay (n = 3). *Controls:* In CN1, sodium taurodeoxycholate from POSTAN was replaced with sodium 10-undecene-1-sulfonate (a sulfonated, non-cholesterol compound), whereas in CN2 it was replaced with an α-tocopherol derivative (a non-sulfonated, non-cholesterol compound).

**Figure S5. In vivo safety evaluation of POSTAN via daily intranasal administration.** Healthy neonatal mice were administered 10 µL of POSTAN nanocarriers (n = 8) or PBS (control) intranasally (IN) once daily for 4 consecutive days (n = 8). Mice were euthanized for tissue collection on the 5th day. Lung tissues were carefully dissected, washed with PBS, and immediately fixed in 10% v/v paraformaldehyde (PFA) overnight for subsequent histological analysis. The slides were then examined by light microscopy. The thickening of the alveolar septum was measured from histological images acquired using a light microscope (20 µm scale bar). Measurements were performed using ImageJ. The measurement tool was calibrated from pixels to micrometers based on the original scale image.

**
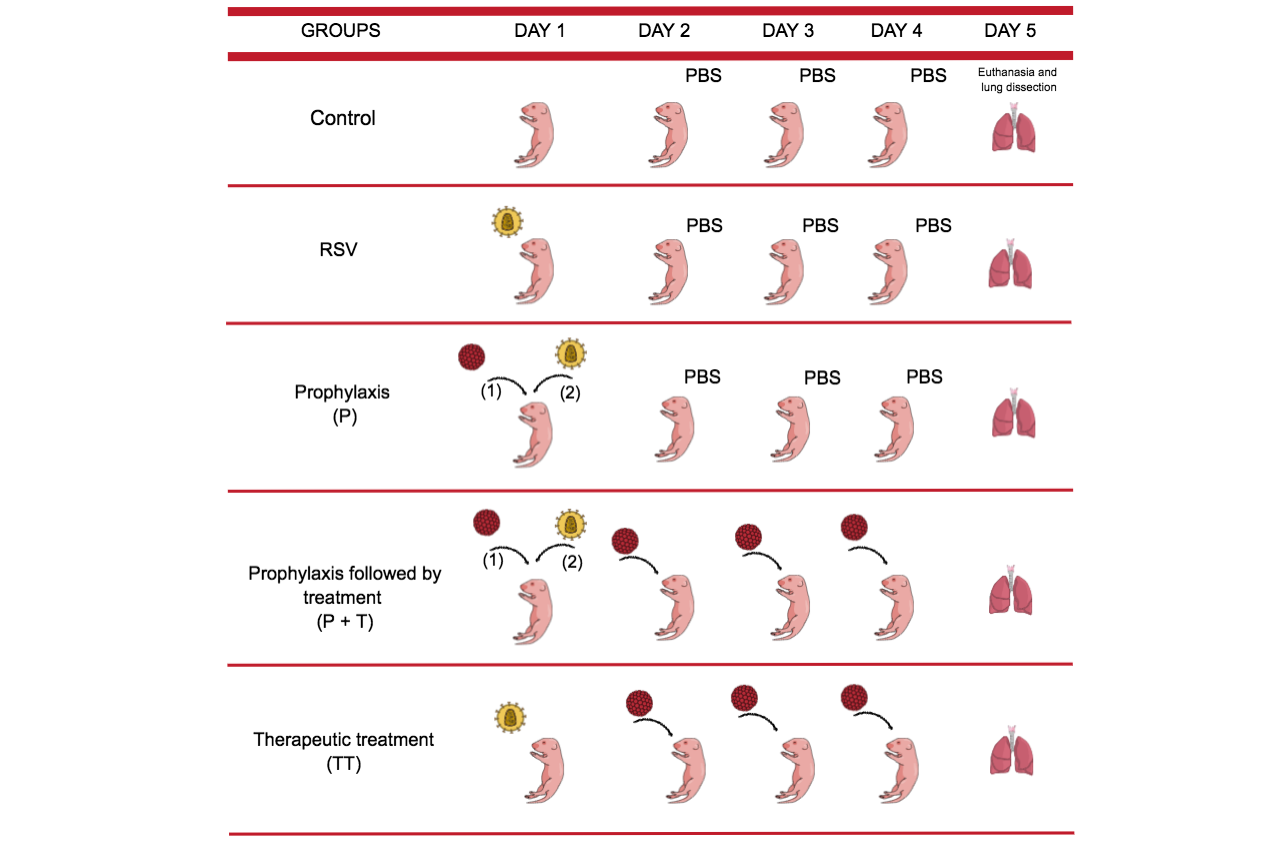
**

**Figure S6. In vivo study model scheme of RSV-A intranasal infection and POSTAN intranasal administration.** Animals were assigned to five groups: (1) Control group: PBS was administered once daily (n = 8); (2) RSV group: PBS was administered once daily starting 24 hpi (n = 8-12); (3) Single prophylaxis dose group (P group): A single dose of POSTAN was administered 15-25 minutes before viral infection, followed by daily PBS administration (n = 8); (4) Prophylaxis and treatment group (P+T group): POSTAN was administered 15-25 minutes before viral infection, followed by daily doses for four days (n = 8); (5) Therapeutic treatment group (TT group): POSTAN was administered once daily for four days, starting 24 hpi (n = 8).


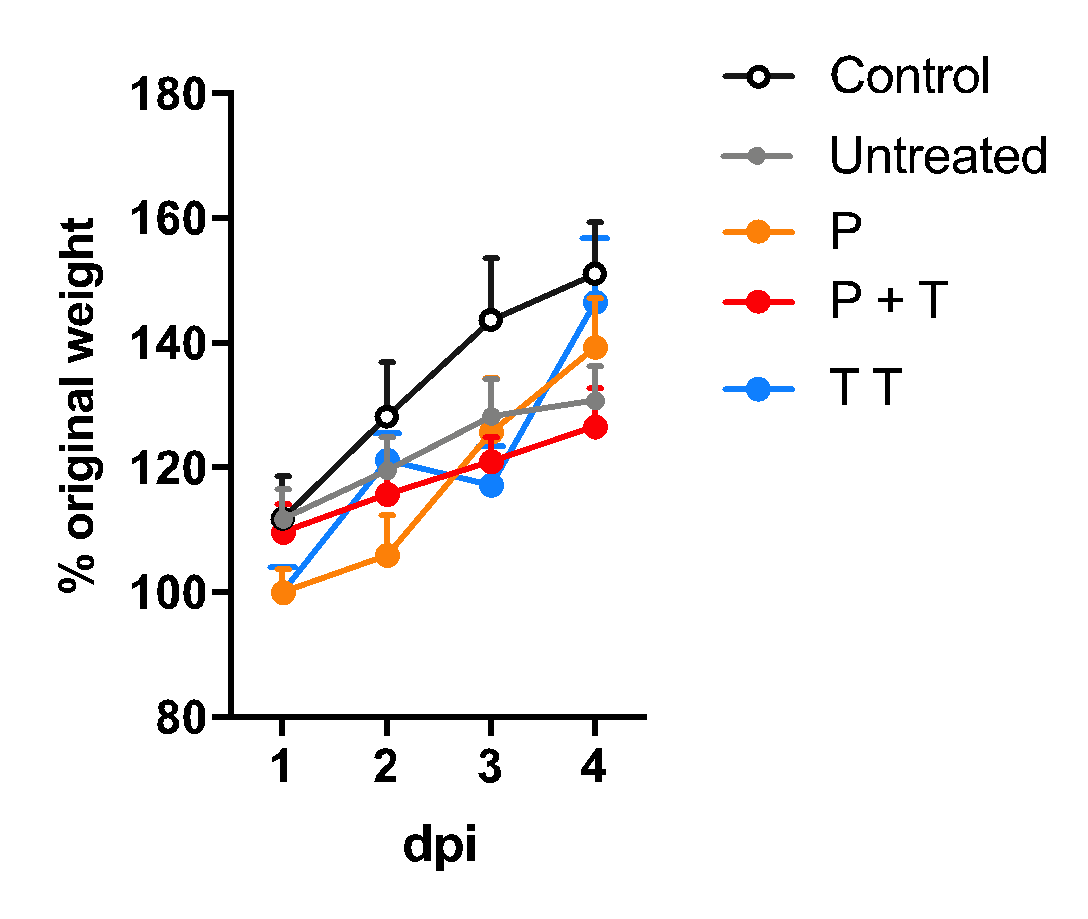


**Figure S7**. **Percentage of weight gain.** The animals were monitored everyday for 4 days beginning of the day throughout the experiment to assess weight gain. Animals were assigned to five groups: (1) Control group: PBS was administered once daily (n = 8); (2) Untreated (RSV group): PBS was administered once daily starting 24 hpi (n = 8-12); (3) Single prophylaxis dose group (P group): A single dose of POSTAN was administered 15-25 minutes before viral infection, followed by daily PBS administration (n = 8); (4) Prophylaxis and treatment group (P+T group): POSTAN was administered 15-25 minutes before viral infection, followed by daily doses for four days (n = 8); (5) Therapeutic treatment group (TT group): POSTAN was administered once daily for four days, starting 24 hpi (n = 8).
